# A meta-analysis of bovine viral diarrhoea virus (BVDV) prevalences in the global cattle population

**DOI:** 10.1038/s41598-018-32831-2

**Published:** 2018-09-26

**Authors:** Bettina Scharnböck, Franz-Ferdinand Roch, Veronika Richter, Carsten Funke, Clair L. Firth, Walter Obritzhauser, Walter Baumgartner, Annemarie Käsbohrer, Beate Pinior

**Affiliations:** 1 Institute for Veterinary Public Health, University of Veterinary Medicine Vienna, Veterinärplatz 1, 1210 Vienna, Austria; 2 Institute of Veterinary Pathology, Justus-Liebig-University, Frankfurter Straße 96, 35392 Giessen, Germany; 3 University Clinic for Ruminants, University of Veterinary Medicine Vienna, Veterinärplatz 1, 1210 Vienna, Austria; 4 Department of Biological Safety, Federal Institute for Risk Assessment (BfR), Diedersdorfer Weg 1, 12277 Berlin, Germany

## Abstract

A random effect meta-analysis was performed to estimate the worldwide pooled bovine viral diarrhoea virus (BVDV) prevalences of persistently infected (PI), viraemic (VI) and antibody-positive (AB) animals and herds. The meta-analysis covered 325 studies in 73 countries that determined the presence or absence of BVDV infections in cattle from 1961 to 2016. In total, 6.5 million animals and 310,548 herds were tested for BVDV infections in the global cattle population. The worldwide pooled PI prevalences at animal level ranged from low (≤0.8% Europe, North America, Australia), medium (>0.8% to 1.6% East Asia) to high (>1.6% West Asia). The PI and AB prevalences in Europe decreased over time, while BVDV prevalence increased in North America. The highest mean pooled PI prevalences at animal level were identified in countries that had failed to implement any BVDV control and/or eradication programmes (including vaccination). Our analysis emphasizes the need for more standardised epidemiological studies to support decision-makers implementing animal health policies for non-globally-regulated animal diseases.

## Introduction

Bovine viral diarrhoea is an important infectious production disease in most cattle-producing countries worldwide^1^. Infections with bovine viral diarrhoea virus (BVDV) have a global economic impact, through high morbidity and mortality rates, increased premature culling and decreased reproductive performance as direct losses^2,3^, as well as the substantial expenditure needed to control BVDV infections as indirect losses^4^. Persistently infected (PI) animals are key to transmitting BVDV as they excrete large amounts of the virus throughout their lives and are unable to develop antibodies to BVDV. In contrast, cattle with transient infections (TI) are less important for disease transmission, often exhibit mild clinical signs, and excrete the virus for only a short period (~14 days)^5^. As both PI and TI animals are viraemic (VI), it is necessary to perform two antigen tests at an interval of at least three weeks in order to distinguish PI from TI animals^6^. Alternatively, one stage testing can be performed with the assistance of epidemiological data^7^ or by using specific diagnostic test methods such as immunohistochemistry (IHC) on skin biopsy specimens^8^. In general, two classifications of diagnostic test methods for BVDV can be distinguished^9,10^: those determining the virus (indicating an active infection) and those detecting antibodies against BVDV (indicating a previous infection). Antibody-positive (AB) animals have been either naturally infected with BVDV, which often leads to lifelong seropositivity^11^ or have been vaccinated. As no marker vaccines for BVDV are yet available^12^, immunological tests cannot routinely differentiate between vaccinated and naturally infected animals, and this has been shown to be a possible cause of concern when interpreting AB test results at a regional or national level^10,13^.

A study of European countries between 1974–1995 by Houe^14^ determined that the PI prevalence at animal level ranged from 0.5 to 2% and that AB prevalence varied between 60 and 85%. The variation in reported BVDV prevalences is known to be influenced by many external factors such as sampling period^11,15^, production type^7^, age of sampled animals^16^, use of BVDV vaccines^11,17,18^, testing of animals with or without clinical signs^14^, implemented control and/or eradication programmes^1,17,19^, and different applied diagnostic methods^6,20^.

To the authors’ knowledge, the data presented here are the first meta-analysis of the prevalences of BVDV infections (i.e., PI, VI and AB) in the global cattle population. The data presented here aim to assist veterinary authorities in the listing and priority categorization of a non-globally-regulated animal disease according to Animal Health Laws (such as EU Regulation 2016/429)^21^. The assessment of BVDV prevalences is of particular importance to veterinary authorities as it can increase the visibility of the worldwide (heterogeneous) distribution of BVDV, and the disease’s potential global economic impact. Furthermore, our study aimed to support policy makers in the decision-making process, if, and when, harmonisation of BVDV mitigation measures and notifications is required. Therefore, the objectives of this study were i) to determine the geographical distribution of BVDV infections; ii) to predict the temporal development of BVDV positive infections in different United Nations (UN) regions; iii) to identify potential sources of heterogeneity among the study outcomes by performing a weighted-stratified meta-analysis of pooled prevalences, including countries by UN regions, sampling periods, production systems, age groups, vaccination status, clinical signs, control and/or eradication programmes, and diagnostic methods; iv) to analyse the explained variance of these factors on the worldwide pooled BVDV prevalences and v) to identify knowledge and standardization gaps in potential influencing factors regarding BVDV prevalences.

## Results

**Figure 1:**
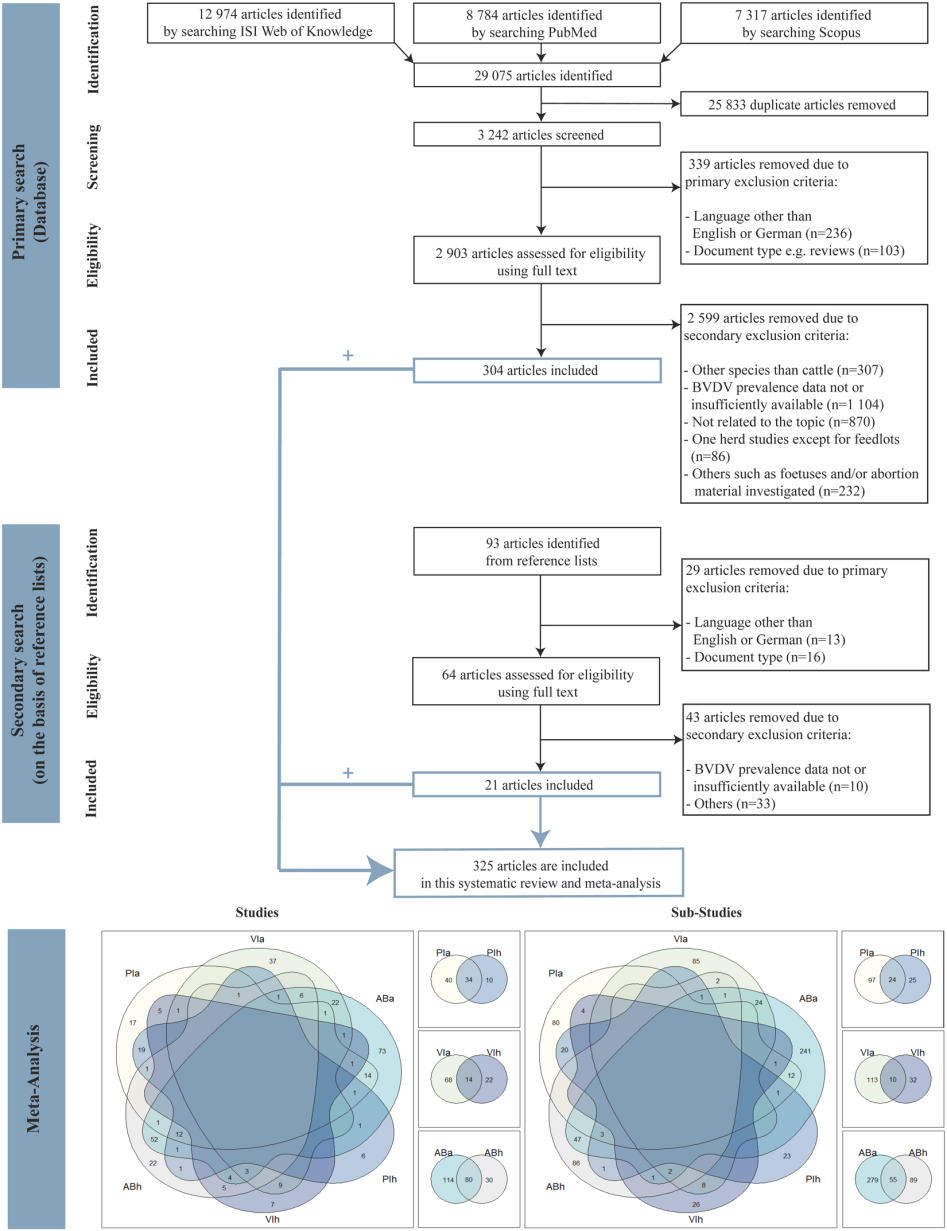
Flow chart of studies incorporated in the systematic review and meta-analysis. PIa = persistent infected animals; VIa = viraemic animals; ABa = antibody-positive animals; PIh = persistent infected herds; VIh = viraemic herds; ABh = antibody-positive herds.

In total, 325 studies conducted in 73 different countries (covering 10 UN regions) for the period 1961–2016 were included in the meta-analysis (Fig. 1). The majority of the studies was performed at regional level (77.02%), followed by national level (13.04%) and farm level (9.94%) (see explanation in Table 1). Overall, 84 studies determined PI prevalences, 104 studies analysed VI prevalences and 224 studies recorded AB prevalences. It should be noted that, as a study may have included more than one prevalence type, the sum of the number of studies which covered PI, VI, and AB prevalences is not identical to the total number of studies (Fig. 1).

**Table 1:** Analysed criteria of the BVDV prevalence studies and summary of these criteria in sub-groups in the meta-analysis and multivariate regression analysis.

Category	Systematic review	Meta-analysis and uni-vs. multivariate regression analysis^a^
Prevalence	Prevalence data, i.e., percentage and/or the total number of tested and positively tested persistently infected (PI), and/or viraemic (VI) and/or antibody-positive (AB) animals/herds were recorded. If only percentage data were available, then the number of infected animals/herds was extrapolated. N.B. the term BVDV prevalence covers PI, VI and AB prevalence at animal or herd level.	Number of tested BVDV positive animals/herds were included in the uni- and multivariate regression model and included as a weighted measure in the meta-analysis. (1) PI animals/herds: This category covered all animals that have been tested twice antigen (AG) positive within a certain period. In a small number of studies, PI prevalence data were published for one-stage testing or were tested AB-negative and AG-positive. If the timing of testing was such that TI could be excluded (e.g., animals tested after birth before colostrum consumption) or specific diagnostic methods were used such as IHC^b^ testing of tissue samples, animals were further processed as PI instead as VI, although these animals were tested only once. N.B. If at least one PI animal was identified in a herd, the herd was classified as a PI herd. (2) PI/VI animals/herds: If information regarding PI and VI animals was simultaneously available i.e., only some of the VI animals were retested, we only considered the PI prevalence data to avoid double counting of information e.g., with regard to vaccination status of herds or implemented BVDV programmes. In case, that all animals were tested and both test results were negative, we processed these data further as PI prevalence. (3) VI animals/herds: Includes animals tested only once (with the exceptions mentioned under category (1) and (2)) or which were proven to have been TI animals/herds. (4) AB-positive animals/herds: Includes BVDV prevalence data of seropositive infected animals/herds or vaccinated animals.
Country/UN region	Country described the area where cattle were tested. Countries were summarised in respective UN regions (Europe, Australia, West Asia, East Asia, South Asia, North America, South America, Central America, North Africa and Sub-Saharan Africa).	Individual countries assigned to the particular UN region were included.
Period	Date of sampling and the study begin was recorded. If both date of sampling and study begin were not available, this was stated as “not specified”. Additionally, the publication year of the study was recorded to perform Forest Plots.	The sampling periods were summarised in four time periods (before 1992, 1992–2001, 2002–2016, not specified).
Production system	Production systems were categorised into dairy, beef, mixed (i.e., dairy, beef, breeding in mixed populations and others such as stock bulls etc.) and not specified (i.e., cattle).	Production systems were categorised into dairy, beef, mixed and not specified.
Age group	Depending on the age of the sampled animals, the prevalence data should be interpreted with caution as, e.g., calves could be tested AB-positive due to maternal antibodies. If information about the age of animals was not provided, this was stated as “not specified”.	The different ages of the animals were summarised into four age groups: ≤6 months; >6 months; animals in both age groups were classified as “mixed”; and if no age group was available the study was classified in the category “not specified”.
Vaccination	Information on whether the animals/herds tested were vaccinated or not were collected. In case some animals in the herd were vaccinated, the herd was considered vaccinated if more than 10% of animals received immunisation against BVDV. If information regarding vaccination status was not available, the studies were assigned in the category “not specified”.	The vaccination status of the animals and herds was classified into three groups: vaccinated (Yes); not vaccinated (No) and “not specified”.
BVD clinical signs	Clinical signs related to infection such as diarrhoea, respiratory symptoms, reproductive disorders, oral lesions, fever, inflammation of the gastrointestinal mucosae were recorded. If no information regarding clinical signs was provided in the studies, these studies were assigned to the category “not specified”.	The animals and herds were classified into three groups: clinical signs (Yes); no clinical signs (No); and if no information was available the study was classified in the category “not specified”.
BVDV programme	Information regarding control and/or eradication activities were recorded. If no information regarding BVDV programmes was provided, this was stated as “not specified”. BVDV control and/or eradication programmes covered voluntary and/or compulsory BVDV programmes at herd, regional or national level. Control and eradication programmes differ in the degree of disease reduction^10^, such as control efforts aiming to reduce BVDV prevalence to a relatively low level, while eradication measures aim to provide a continued absence of BVDV^10,19^. In this study, control programmes did not cover vaccination.	Information about the implemented BVDV programmes were included in the meta-analysis. The information of the BVDV control and/or eradication programmes were distinguished as follows: animals/herds included in BVDV programmes were assigned to the category “Yes”; animals/herds not included in BVDV programmes were assigned to the category “No” and if no information was available, this was covered in the category “not specified”.
Diagnostic method	Types of diagnostic methods used were collected and wherever available the corresponding sensitivity and specificity were recorded.	The different applied diagnostic methods were classified as follows: (1) Direct detection methods comprised IHC^b^ and IFT^c^; (2) RT-PCR^d^ covered RT-PCR, qPCR^e^, PCR^f^, real-time-RT-PCR; (3) AG ELISA covered AG ELISA^g^ or AC ELISA^h^; (4) Cell culture-based systems covered virus isolation with immunoperoxidase, fluorescent antibody or interference test; (5) Mixed covered combinations of diagnostic methods for screening and confirmation of laboratory results (two-test strategy); (6) AB ELISA^i^ covered AB ELISA, Cell bound immunoassay; (7) NT^j^ covered serum-, virus-, microneutralisation assays; (8) Mixed covered both AB ELISA and NT^j^; (9) Other covered e.g., IFT^c^ and complement fixation tests.
Sample material	Sample materials such as tissues from living or dead animals except for foetuses and abortion material were recorded. Abortion materials were not incorporated into our analyses because they were considered as unsuitable for diagnostic testing^9^.	The different sample materials were included in the uni- and multivariate regression analysis and were classified as follows: (1) Blood e.g., whole blood, buffy coat, sera; (2) Blood and milk; (3) Blood and other; (4) Blood and tissue sample; (5) Blood, tissue sample and other; 6) Milk; (7) Tissue sample and other; (8) Tissue sample e.g., ear notch biopsy, brain tissue, spleen, tracheal lymph node, cerebellar tissue; (9) Other e.g., nasal swab, trans-tracheal aspirate, cerebrospinal fluid, rectal swabs.
Level	The studies were categorised into three levels. (1) Farm level: studies covered two to five herds; (2) Regional level: either a certain area/region in a country was sampled or more than five herds (with the exception of feedlot studies, where animals from different areas of a country were investigated); (3) National level: the total cattle population or a representative subpopulation of the total population of one country was tested. Studies not assignable to one of these categories were included in the category “not specified”.	The levels were included in the uni- and multivariate regression analysis.
Genotype	Information regarding to BVDV genotypes 1, 2 and HoBi-like strains was collected.	The genotypes were not included in the meta-analysis and also not incorporated in the uni- and multivariate regression analysis due to insufficient data.

^a^Unless otherwise stated, all categories were included in both meta-analysis and multivariate regression analysis.

^b^Immunohistochemistry.

^c^Immunofluorescence tests.

^d^Reverse transcription polymerase chain reaction.

^e^Quantitative polymerase chain reaction.

^f^Polymerase chain reaction.

^g^Antigen enzyme-linked immunosorbent assay.

^h^A polyclonal antibody-based antigen-capture ELISA.

^i^Antigen enzyme-linked immunosorbent assay.

^j^Neutralisation assays.

**Figure 2:**
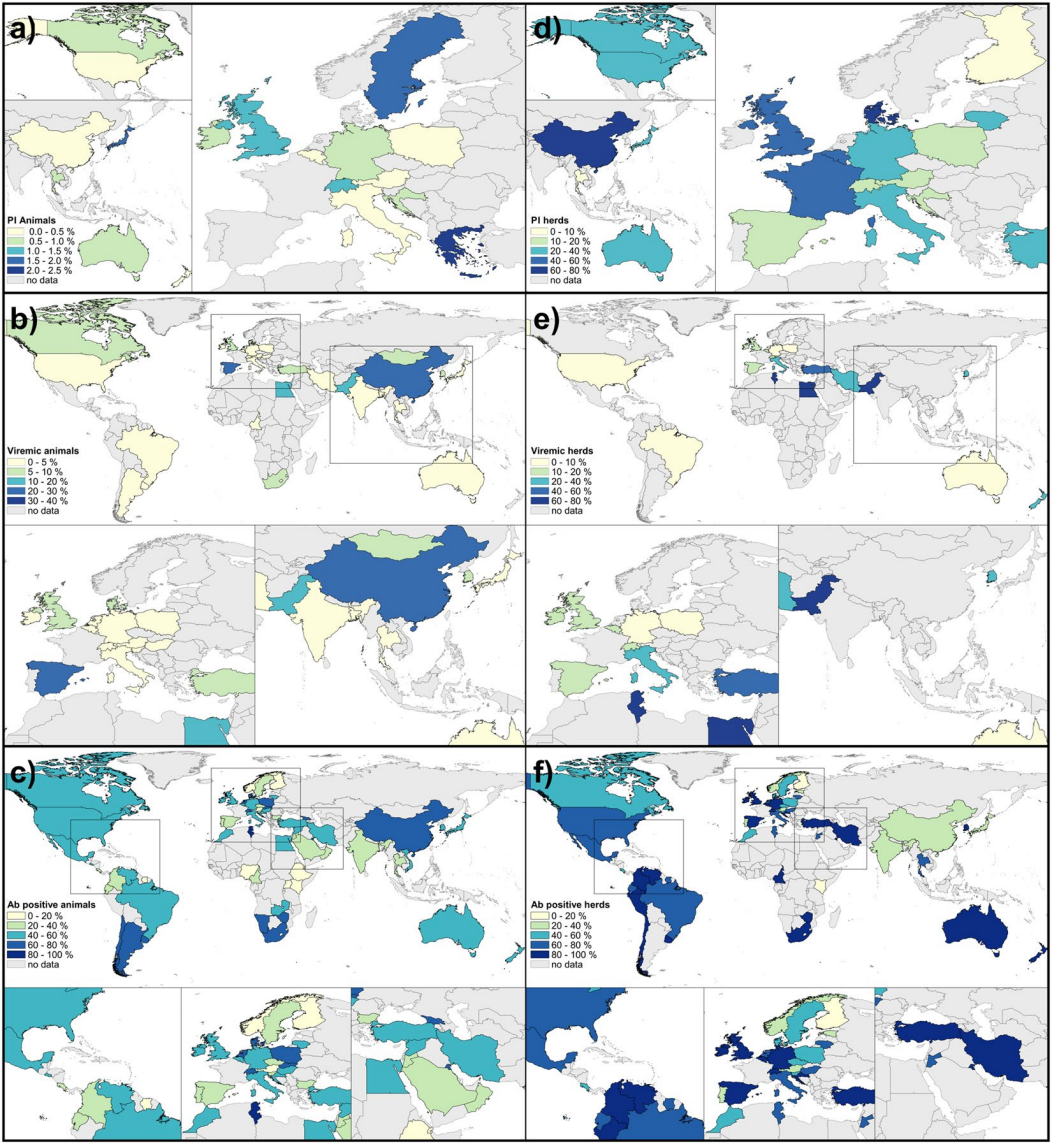
Pooled prevalences per country for the entire study period. Maps on the left show the prevalences at animal level (**a** = PI; **b** = VI; **c** = AB) while those on the right illustrate the prevalences at herd level (**d** = PI; **e** = VI; **f** = AB). N.B. The intersection between the number of studies at animal level and herd level is provided in Fig. 1. The temporal development of BVDV prevalences for each country is provided in the Forest Plots (Supplementary Figs S1–S3).

Overall, 6.5 million animals and 310,548 herds were tested for BVDV infections worldwide. The majority of these tests was performed at animal level to identify persistent infections (75.07%; 4.9 million tested animals) and at herd level to detect AB-positive animals (69.59%; 216,105 herds). By location, prevalence assessments for BVDV infections were performed most frequently in Europe (PI animal: 48.76%, n = 59; PI herd: 40.82%, n = 20; AB animal: 45.51%, n = 152; AB herd: 53.47%, n = 77), followed by North America (PI animal: 26.45%, n = 32; PI herd: 26.53%, n = 13; AB animal: 10.78%, n = 36; AB herd: 9.72%, n = 14; see Tables 2–4). The geographical distribution of pooled BVDV prevalences at animal and herd level per country is shown in Fig. 2. In addition, the development of BVDV prevalences per country over time is provided in Supplementary Forest Plot Figures S1–S3.

**Table 2:** Meta-analysis of studies reporting the prevalence of PI animals.

	No. prevalence inputs	Sample size (No. animals)	Weighted mean estimate	Confidence Interval (95%)	I^2^ (%)
Overall	121	4,904,119	0.77	(0.59–0.97)	99.73
**Subgroup** ^**a**^					
**UN Region**					
Europe	59	4,698,632	0.76	(0.50–1.08)	99.89
North America	32	106,647	0.50	(0.29–0.75)	94.02
East Asia	11	58,188	1.07	(0.36–2.08)	98.06
West Asia	6	2,309	2.61	(1.36–4.23)	72.52
Australia	13	38,343	0.46	(0.22–0.76)	38.71
**Period**					
1980–1991	6	4,361	1.85	(0.65–3.59)	86.50
1992–2001	25	92,689	0.90	(0.53–1.36)	96.64
2002–2016	59	4,765,501	0.36	(0.23–0.51)	99.72
Not specified	31	41,568	1.58	(1.03–2.23)	94.59
**Production system**					
Beef	28	201,743	0.35	(0.20–0.54)	95.88
Dairy	51	85,786	1.13	(0.82–1.49)	91.86
Mixed	19	2,660,173	1.10	(0.64–1.67)	99.87
Not specified	23	1,956,417	0.51	(0.13–1.08)	99.93
**Age group**					
≤6 months	23	2,282,771	0.70	(0.27–1.29)	99.93
>6 months	13	144,800	0.93	(0.28–1.88)	99.29
Mixed	32	1,624,080	0.71	(0.41–1.08)	98.54
Not specified	53	852,468	0.82	(0.57–1.10)	98.67
**Vaccination**					
Yes	19	139,423	0.24	(0.15–0.36)	87.46
No	34	2,547,568	0.54	(0.28–0.86)	99.76
Not specified	68	2,217,128	1.08	(0.79–1.41)	99.70
**Clinical signs**					
Yes	15	44,514	2.22	(1.26–3.43)	97.66
No	5	25,597	1.45	(0.51–2.81)	97.65
Not specified	101	4,834,008	0.55	(0.41–0.72)	99.67
**Programme**					
Yes	34	4,707,613	0.47	(0.25–0.74)	99.90
No	3	2,284	0.59	(0.00–1.77)	76.20
Not specified	84	194,222	0.94	(0.69–1.22)	96.08
**Diagnostic method** ^**b**^					
Direct detection method	6	18,165	0.97	(0.28–2.00)	96.69
RT-PCR	12	184,472	0.32	(0.02–0.83)	99.24
AG ELISA	49	1,279,649	0.75	(0.50–1.04)	99.47
Cell culture-based system	21	40,848	1.42	(0.75–2.28)	96.59
Mixed	33	3,380,985	0.57	(0.33–0.86)	99.82

^a^All p-values except for Australia (p = 0.01) were ≤0.01 and were calculated using the Cochran’s Q-test.

^b^The description of the diagnostic methods is provided in Table 1.

**Table 3:** Meta-analysis of studies reporting the prevalence of VI animals.

	No. prevalence inputs	Sample size (No. animals)	Weighted mean estimate	Confidence Interval (95%)	I^2^ (%)
Overall	123	1,169,170	4.37	(3.10–5.82)	99.85
**Subgroup** ^**a**^					
**UN Region**					
Europe	49	1,093,215	2.38	(1.28–3.75)	99.87
North America	29	49,605	6.95	(3.49–11.38)	99.59
South America	3	9,638	1.13	(0.00–3.96)	91.47
East Asia	10	3,716	11.22	(3.74–21.77)	98.53
West Asia	13	3,399	5.35	(1.43–11.24)	97.19
South Asia	10	4,219	1.80	(0.22–4.44)	94.65
Australia	2	1,627	1.35	(0.00–4.54)	78.37
North Africa	2	268	17.88	(5.12–35.83)	89.47
Sub-Saharan Africa	5	3,483	4.87	(0.00–18.54)	99.33
**Period**					
1982–1991	23	18,232	2.82	(1.15–5.06)	97.85
1992–2001	22	21,989	7.78	(3.33–13.75)	99.44
2002–2016	45	1,119,545	3.04	(1.44–5.11)	99.94
Not specified	33	9,404	5.80	(3.23–9.00)	96.67
**Production system**					
Beef	20	8,440	7.80	(2.83–14.76)	98.88
Dairy	46	17,023	2.26	(1.10–3.73)	96.15
Mixed	23	694,310	7.37	(4.48–10.86)	99.48
Not specified	34	449,397	4.03	(1.82–6.96)	99.86
**Age group**					
≤6 months	20	1,016,089	4.18	(1.91–7.18)	99.94
>6 months	27	24,058	1.37	(0.52–2.50)	96.13
Mixed	21	54,833	2.08	(0.52–4.40)	99.38
Not specified	55	74,190	7.56	(4.82–10.81)	99.52
**Vaccination**					
Yes	8	3,316	7.84	(1.20–18.89)	98.46
No	46	691,269	2.38	(0.99–4.22)	99.13
Not specified	69	474,585	5.68	(3.84–7.81)	99.73
**Clinical signs**					
Yes	66	36,196	7.11	(4.81–9.79)	98.65
No	8	3,840	5.24	(1.39–11.04)	93.25
Not specified	49	1,129,134	1.56	(0.68–2.69)	99.88
**Programme**					
Yes	15	1,067,930	1.85	(0.30–4.32)	99.96
No	3	1,526	1.61	(0.00–6.12)	92.62
Not specified	105	99,714	4.94	(3.44–6.65)	99.14
**Diagnostic method** ^**b**^					
Direct detection method	13	3,051	9.30	(3.37–17.54)	97.36
RT-PCR	31	21,802	5.95	(2.98–9.74)	98.79
AG ELISA	29	361,629	3.00	(1.31–5.24)	98.99
Cell culture-based system	26	18,202	1.85	(0.61–3.59)	97.19
Mixed	24	764,486	5.09	(2.14–9.08)	99.91

^a^All p-values except for Australia (p = 0.03) were ≤0.01 and were calculated using the Cochran’s Q-test.

^b^The description of the diagnostic methods is provided in Table 1.

**Table 4:** Meta-analysis of studies reporting the prevalence of AB-positive animals.

	No. prevalence inputs	Sample size (No. animals)	Weighted mean estimate	Confidence Interval (95%)	I^2^ (%)
Overall	334	459,095	49.20	(46.14–52.25)	99.76
**Subgroup** ^**a**^					
**UN Region**					
Europe	152	343,629	48.38	(43.65–53.13)	99.86
North America	36	23,737	53.35	(41.94–64.60)	99.67
Central America	8	3,593	41.00	(24.60–58.48)	99.06
South America	27	17,743	53.43	(44.19–62.57)	99.27
East Asia	15	11,017	40.88	(28.86–53.46)	99.40
West Asia	37	11,257	51.06	(43.68–58.42)	98.35
South Asia	15	11,598	50.82	(37.35–64.22)	99.40
Australia	17	20,326	52.60	(43.01–62.09)	99.35
North Africa	8	1,942	53.97	(40.92–66.74)	96.12
Sub-Saharan Africa	19	14,253	42.06	(27.42–57.43)	99.69
**Period**					
1961–1991	48	35,857	48.73	(41.20–56.28)	99.33
1992–2001	100	93,611	46.82	(41.38–52.29)	99.63
2002–2016	72	262,829	46.23	(39.12–53.41)	99.92
Not specified	114	66,798	53.38	(48.28–58.44)	99.40
**Production system**					
Beef	40	63,210	45.14	(34.36–56.15)	99.87
Dairy	168	79,367	51.76	(47.67–55.84)	99.24
Mixed	42	96,219	48.52	(39.63–57.47)	99.86
Not specified	84	220,299	46.34	(40.64–52.09)	99.84
**Age group**					
≤6 months	33	5,869	47.68	(37.17–58.29)	98.42
>6 months	64	176,293	49.05	(41.82–56.31)	99.86
Mixed	93	66,863	48.89	(43.01–54.78)	99.55
Not specified	144	210,070	49.81	(45.37–54.26)	99.75
**Vaccination**					
Yes	21	28,653	66.04	(52.24–78.60)	99.80
No	177	272,973	45.19	(41.29–49.12)	99.74
Not specified	136	157,469	51.76	(46.89–56.60)	99.72
**Clinical signs**					
Yes	93	32,828	52.26	(46.58–57.92)	99.03
No	25	15,077	36.85	(28.51–45.59)	99.08
Not specified	216	411,190	49.35	(45.48–53.21)	99.83
**Programme**					
Yes	27	251,497	29.52	(20.58–39.31)	99.96
No	16	9,651	45.47	(35.69–55.43)	98.83
Not specified	291	197,947	51.34	(48.09–54.58)	99.51
**Diagnostic method** ^**b**^					
AB ELISA	171	352,277	45.67	(41.42–49.96)	99.84
NT	146	94,929	54.26	(49.90–58.58)	99.41
Mixed	7	2,562	38.38	(16.00–63.69)	99.32
Other	10	9,327	43.63	(23.04–65.42)	99.73

^a^All p-values were ≤0.01 and were calculated using the Cochran’s Q-test.

^b^The description of the diagnostic methods is provided in Table 1.

**Figure 3:**
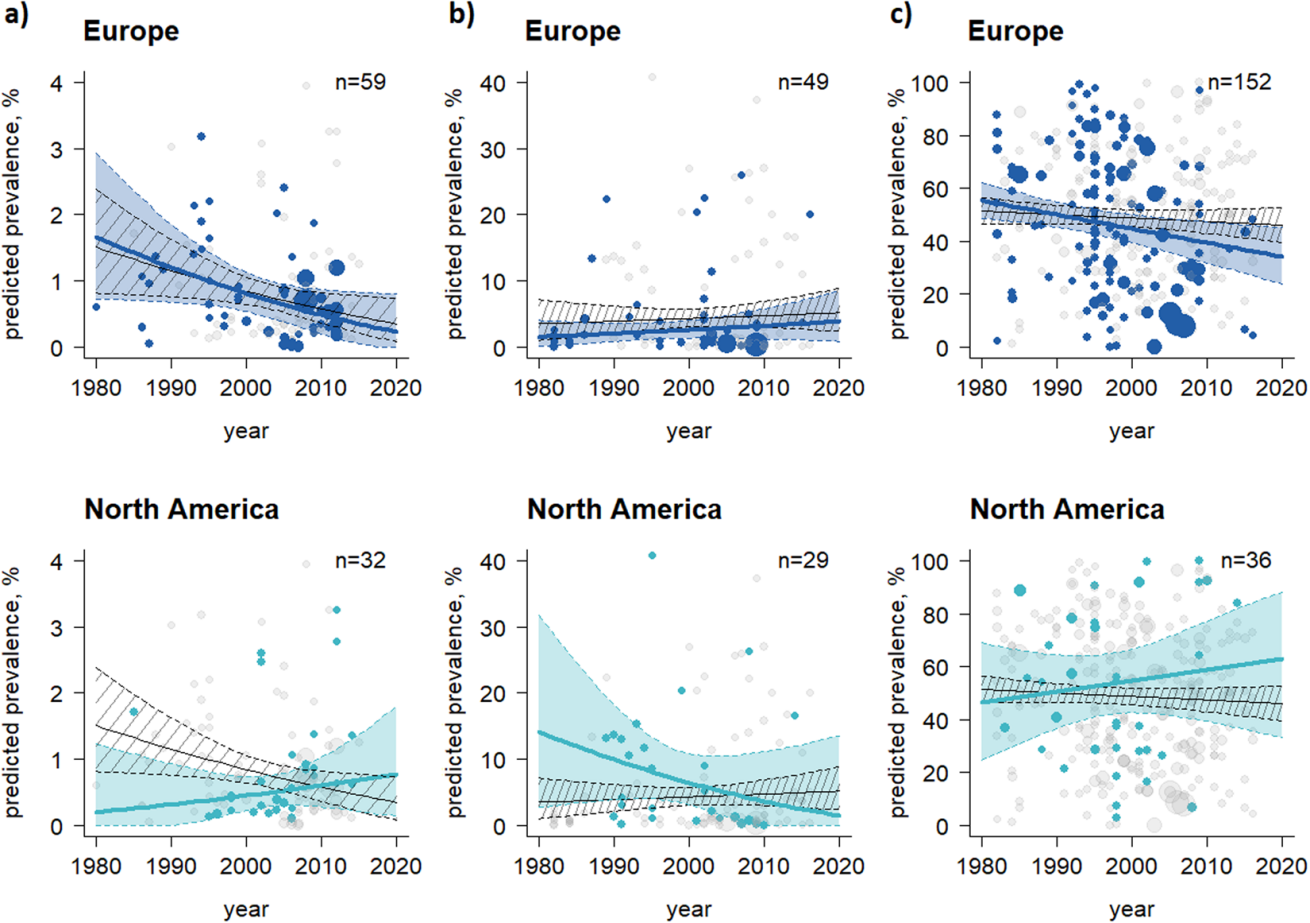
Temporal trend analysis at animal level stratified by UN region, period and type of BVDV prevalences. (**a**) PI animals, (**b**) VI animals, and (**c**) AB-positive animals. The thin black lines represent the mean prevalence estimates of all 10 UN regions with the corresponding 95% CI (black shaded area) and individual prevalence points of studies in the 10 UN regions (grey dots) during the period observed. The coloured lines, areas and dots highlight the prevalence estimates for Europe and North America. The more prevalence estimates available at a certain time, the wider the dots. The predictions were calculated for all 10 UN regions together (black lines and shaded areas), as well as individually for Europe and North America (coloured) at the period, where the last dot is shown i.e., the PI prevalence in Europe and North America was predicted until 2020 based on data reported before 2013 and 2015, respectively. UN regions that had less than 15 sub-studies were not analysed individually. The abbreviation n represents the total number of sub-studies considered in Europe and North America (Tables 2–4). UN regions that recorded PI prevalences at animal level were used as a baseline for the UN regions that recorded VI and AB prevalences.

**Figure 4:**
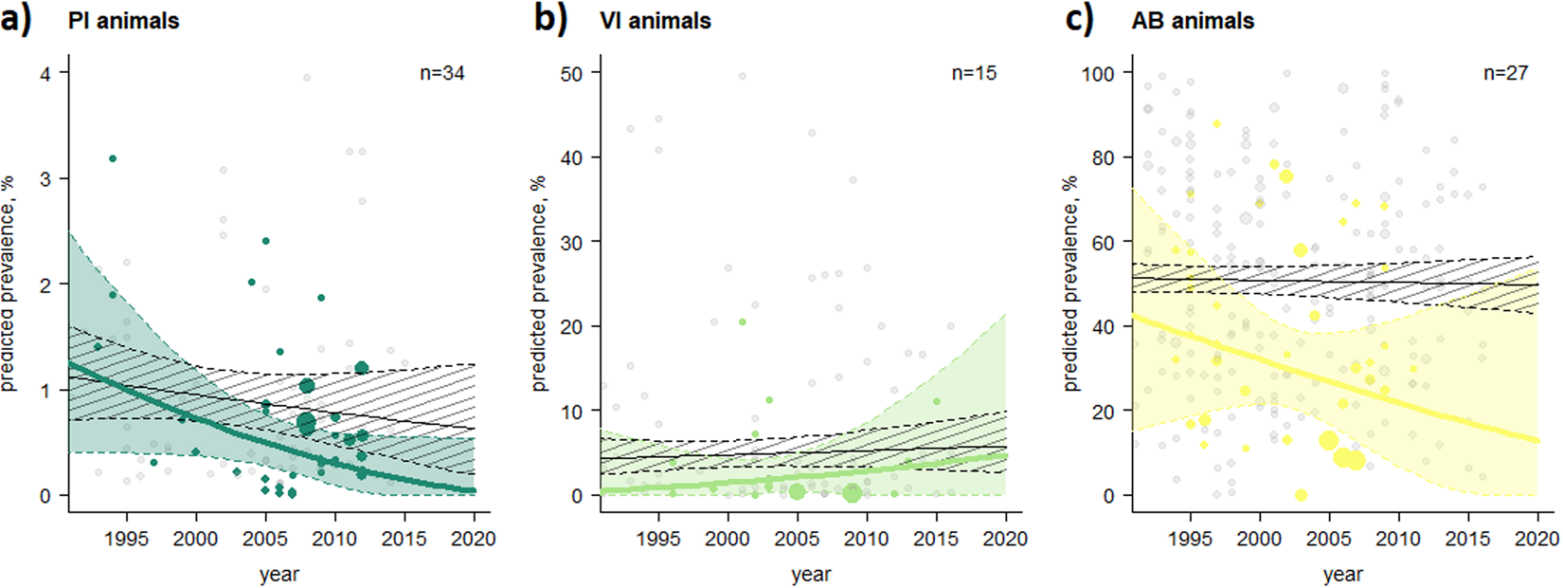
Temporal trend analysis at animal level stratified by BVDV control and/or eradication programmes (Yes/No and not specified), period and type of BVDV prevalences. (**a**) PI animals, (**b**) VI animals, and (**c**) AB-positive animals. The thin black lines represent the mean prevalence estimates of studies without/not specified BVDV control and/or eradication programmes with the corresponding 95% CI (black shaded area) and individual prevalence points of these studies (grey dots) during the period observed. The coloured lines, areas and dots highlight the prevalence estimates of studies with BVDV control and/or eradication programmes. The more prevalence estimates available at a certain time, the wider the dots. The abbreviation n represents the number of studies with BVDV control and/or eradication programmes (coloured dots). N.B. the period started in 1992 because the first BVDV control and/or eradication programme was implemented at that time.

The pooled worldwide PI prevalence decreased from 1.85% (95% CI: 0.65–3.59) to 0.36% (95% CI: 0.23–0.51) between 1980 and 2016 at animal level (Table 2) and declined from 42.36% (95% CI 23.91–61.83) to 18.88% (95% CI: 8.79–31.17) at herd level (Supplementary Table S1). A reduction was also observed of VI prevalences at animal and herd level (Table 3, Supplementary Table S2). The highest mean pooled PI prevalence was identified in dairy production systems, in animals aged >6 months, in non-vaccinated herds, in animals with clinical signs, in animals not enrolled in control and/or eradication programmes, and those tested with cell culture-based systems (Table 2). The temporal trend analysis predicted an overall decrease in PI animals in Europe and an increase in North America (Fig. 3). In contrast to PI prevalences, the mean pooled seroprevalence (AB prevalence) over all UN regions and during the study period remained relatively constant at animal level (ranging from 48.73% to 46.23%) and at herd level (from 67.01% to 66.08%). However, an increase of AB prevalences was predicted in North America, while a decrease was projected for Europe (Fig. 3). The AB prevalence determined for animals/herds not participating in control and/or eradication programmes was double that of those animals/herds under control systems (Table 4; Supplementary Table S3). Figure 4 shows the success of BVDV control and/or eradication programmes, with regard to reductions in BVDV prevalences.

Multicollinearity was determined between the factors “BVDV control and/or eradication programmes” and “countries” (ranged from 0.55 to 0.88). Consequently, BVDV programmes were excluded from our meta-regression analyses. The explained variance of the remaining factors on the worldwide pooled BVDV prevalence is shown in Table 5. The regression test for funnel plot asymmetry indicates no significant publication bias in AB prevalences (z = 0.81; *P* = 0.41), while a publication bias was determined for PI (z = 5.97; *P* < 0.001) and VI prevalences (z = 4.36; *P* < 0.001).

**Table 5:** Uni- and multivariate meta-regression model stratified by factors and BVDV prevalences at animal and herd level.

Factors	Animal level (R^2^ univariate)			Herd level (R^2^ univariate)		
	PI	VI	AB	PI	VI	AB
No. of tested animals/herds	—	—	2.1	16.8	—	10.8
Country	28.0	13.6	17.0	—	—	61.2
Period	23.0	3.3	—	8.4	—	—
Production system	12.6	6.1	—	—	—	—
Age group	—	10.8	—	—	24.4	—
Vaccination	10.0	4.0	3.2	4.3	6.0	5.5
Clinical signs	19.5	12.3	1.2	—	18.1	—
Diagnostic method	5.6	4.4	1.8	—	—	—
Sample material	1.9	28.9	3.3	—	17.0	4.1
Level	—	—	—	14.2	20.2	8.5
R^2^ (multivariate)	55.6	63.0	26.2	40.1	35.7	58.9

## Discussion

In today’s globalised world, with international trade in live animals commonplace, it is essential to ensure that diseases are not transported across borders^17,22,23^. The analysis presented here revealed a wide variation of BVDV prevalences both within, and between, UN regions (Supplementary Figs S1–S3), and exposed the incomplete picture of the epidemiological situation of BVDV for a number of countries (Fig. 2). Across all UN regions, we observed a decrease in PI prevalences and a relatively stable level of AB prevalences over time (Tables 2 and 4). Our temporal analysis predicted a decrease in PI prevalences in Europe during the projected period (Fig. 3), which appears to be related to BVDV control and/or eradication programmes. Our analyses show that countries which have implemented control and/or eradication programmes have an, on average, 1.5 times lower pooled BVDV prevalence at animal level and herd level compared to countries without intervention measures (Tables 2–4; Supplementary Tables S1–S3; with the exception of VI prevalence at animal level as the animals in the category “no programme” were three times older than those “within a programme”. Consequently, the probability of detecting the virus decreases because the likelihood that the animal had previously been in contact with the virus and developed an immune response (i.e., produced AB) increases). These results are also shown in Fig. 4 and are in line with prevalence surveys from the late 1970s and 2000s^17^.

The current BVDV epidemiological situation demonstrates that several countries that implemented BVDV intervention programmes have successfully reduced PI prevalences at animal level, e.g., Austria (decreased from 0.13% in 2006; n = 2600 PI animals on approximately 1700 farms; to 0.00% in 2017; n = 3 PI animals on 3 farms, only one of these cases was a new infection)^24^, Denmark (decreased from 1.40% in 1988 (this prevalence was not determined at national level)^25^ to 0.00% in 2014), Germany (proportion of newborn PI calves decreased from 0.48% in 2011 to 0.01% in 2017)^26^, Ireland (proportion of newborn PI calves decreased from 0.77% in 2013 to 0.12% in 2018)^27^, Switzerland (proportion of newborn PI calves decreased from 1.4% in 2008 to 0.02% in 2012)^28^, while Sweden, Finland, Norway have completely eradicated BVDV (as of May 2018). The success of such intervention measures has prompted the national veterinary authorities of some countries to revise sampling schemes, changing from control and/or eradication programmes to risk-based surveillance testing strategies such as those carried out in some federal states in Austria (since 2018; only for non-dairy farms), Switzerland (since 2012) and Denmark (since 2006), whereas other countries such as Ireland are currently analyzing the effectiveness of different testing strategies^29^. Although BVDV prevalences in Europe have decreased in the past, premature discontinuation of control efforts should be treated with caution as a seronegative cattle population will be fully susceptible to BVDV. Official international trade restrictions are not currently in place^7^ and the epidemiological situation for this disease is unknown in a number of countries worldwide, resulting in a constant risk of re-introduction of BVDV into disease-free regions. Previous studies have demonstrated that the majority of novel BVDV infections could be effectively reduced if livestock trade would be under more stringent controls^17,30^.

In the present study, the highest PI prevalences at animal level were identified in Western and Eastern Asia (Table 2). These regions have not implemented national control and/or eradication programmes, or vaccination programmes, and studies mainly reported dairy farm testing (71.43% of all tests). Although national compulsory eradication programmes have not been implemented in Australia or North America either, the lowest PI prevalence was observed in these regions. The relatively low PI prevalence at animal level in Australia can primarily be explained by testing of adult cattle, which is unlikely to detect PI animals, vaccination, and (voluntary) testing of traded animals to confirm their freedom from disease. Similarly, in North America, the relatively low proportion of PI animals detected can be explained by the testing of beef rather than dairy herds, implemented voluntary control and/or eradication programmes (such as those in Colorado, Alabama, Georgia, Mississippi, Montana, Oregon, Washington, New York, and the Upper Peninsula of Michigan)^31^ and by BVDV vaccination.

Approximately 80% of the cattle population in North America is vaccinated, and this vaccination rate is estimated to be four times higher than in Europe (20% of the cattle population vaccinated)^32^. This finding is supported by our meta-analysis, as the number of studies assessing BVDV vaccines at animal and herd level was approximately three times higher in North America than in Europe. It is perhaps important to note, however, that a number of countries in Europe (e.g., Austria, Denmark, Finland, Sweden, Norway) do not permit BVDV vaccination. The variety of vaccination strategies applied might explain the inverse predicted development of AB prevalences between North America and Europe (Fig. 3). This may lead to a balance of AB prevalences between all considered UN regions during the analysed period (Table 4; Supplementary Table S3).

The temporal time analysis also predicted a slight increase in pooled PI prevalence in North America until 2020, mainly due to reportedly high prevalences in Canada. Excluding prevalence data for Canada, PI prevalence appears likely to remain constant during the study period (Fig. 3). Thus, the current control strategy of BVDV vaccination in North America seems to maintain the PI prevalence at a relatively low level but is still not suitable to eradicate BVDV. Approximately 10% of animals reach breeding age without immunological protection against BVDV to vaccination in the US^33^. Possible explanations for the difficulty of eradicating BVDV by using vaccines could be that (i) removal of PI animals must be completed before vaccination, because to the authors’ knowledge vaccination as a successful stand-alone strategy to eradicate BVDV had never been reported in the literature, (ii) a critical vaccination coverage rate should be reached to prevent new persistent infections, (iii) farmers using vaccines often neglect to maintain or implement biosecurity measures^31^, (iv) farmers use vaccines incorrectly, e.g., by applying vaccination after insemination, (v) in general, vaccines are not proven to be fully efficient in the prevention of in-utero transmission of the virus^32^, and (vi) the risk of live BVDV vaccine becoming contaminated with other viruses^15^. These aspects may explain why approximately 1.4 times higher BVDV prevalences (without seroprevalences) were observed in studies of vaccination strategies compared to non-vaccination studies. For instance, BVDV programmes using vaccination were found to have a higher PI pooled prevalence (0.44%) at animal level than programmes without vaccination (0.31%). Nonetheless, the highest mean pooled PI prevalences at animal level were identified in countries that did not use vaccines or failed to implement BVDV control and/or eradication programmes. In contrast to our results, Newcomer and colleagues^34^ showed in a meta-analysis a significantly positive effect of vaccines in preventing foetal infections with BVDV. Our analysis differs in the definition of vaccinated herds, the aims and the type and number of studies which were taken into account compared to the study by Newcomer *et al*.^34^. More than half of the studies considered by Newcomer *et al*.^34^ were excluded from our meta-analysis due to insufficient data on the number of tested animals and herds. The positive effect of vaccination against persistent infection at animal level might be mitigated by consideration that 73.68% of the studies applying BVDV vaccines were conducted in beef herds. In general, dairy cattle are managed much more intensively than beef cattle herds and thus the spread of infection is likely different between both production systems^35^. The higher infection risk in dairy production systems (across all UN regions; three times higher PI prevalences were identified in dairy compared to beef herds) might be the result of i) higher frequency of animal contacts in dairy herds which increase the probability of virus transmission (e.g. beef herds are often less intensively farmed than dairy herds^36^, with lower stocking densities and less invasive management, generally leading to an overall lower level of disease), and ii) most beef herds having a seasonal breeding period^31,35^ and a shorter lifespan compared to dairy herds, and thus likely a higher self-clearance rate (i.e., persistent infection is eliminated from the herd without active disease intervention but through natural deaths, slaughter and culling). While some countries, such as Austria, often use dual-purpose cattle as dairy cows, rearing the heifer calves for dairy and the bull calves for beef, many countries have a much more stratified cattle industry. For instance, in U.S., beef production, calves are in permanent contact with their dams and with pregnant cows from the start of the breeding season until weaning at 5 to 7 months of age^35^. By contrast, in dairy systems the U.S., calves are removed from their dams^37^ (and from all potentially pregnant cows) until they are returned to the herd as a pregnant heifer. This could explain why the PI pooled prevalence in U.S. beef production systems (0.41%) was higher than in dairy herds (0.29%), and illustrates an opposite trend to the other UN regions.

With respect to persistent infections, we identified more studies that have tested animals younger than six months than animals older than six months of age. This finding is supported by Sarrazin *et al*.^38^, who reported that detection of PI animals at adult age occurs less frequently. The lifespan of PI animals is most often limited due to a high mortality among these animals dying of mucosal disease, other complications or PI animals being slaughtered due to poor performance^11^. Loneragan and colleagues^39^ pointed out that PI animals are thus overrepresented in dead cattle and the early death of animals prior to testing influences the published PI prevalences^15^. To address the potential limitation of missed PI cattle in dead animals, our meta-analysis also covered studies that tested dead animals. Unfortunately, only four of these types of studies were available. Additionally, maternal antibodies can mask the presence of viral antigens within 28 days after birth which may also lead to false-negative virus isolation and thus to an underestimation of the identified pooled PI prevalence in the age category ≤6 months^40,41^. The generally low number of tested animals aged >6 months, the early death of young animals prior to testing, and the possible masking of viral antigens due to maternal antibodies among younger animals could explain the higher identified pooled PI prevalence of animals aged >6 months compared to animals ≤6 months. To avoid false-negative virus isolation, RT-PCR or IHC from skin biopsies would be suitable to verify the PI status in the presence of colostral antibodies^42^, but Table 2 shows that such diagnostic methods were rarely applied. Furthermore, PCR has been shown to detect BVDV RNA in skin tissue from newborn calves whose mothers were vaccinated with BVDV live vaccines during pregnancy^13^ indicating that such molecular biological techniques are not necessarily failsafe methods. An overview of the advantages and disadvantages of different BVDV testing methods depending on the diagnostic situation are provided in the studies by Larson *et al*.^6^, Houe *et al*.^10^ and Lanyon *et al*.^20^. In the age category 6–24 months, a lower AB prevalence was determined (34.66%; excluding vaccinated animals: 33.72%) than for animals older than two years (56.51%; excluding vaccination 52.22%). This finding is supported by other studies, reporting that BVDV seroprevalence is likely to be higher in older animals as they are more likely to have been exposed to BVDV exponentially over time, and as previously infected animals remain seropositive for the rest of their lives^11,16,38^. Nonetheless, our analysis also demonstrated a relatively high AB prevalence in the age category ≤6 months. Although the majority of these studies (80%) did not cover BVDV vaccines, it cannot be excluded that some of these studies reported false-positive test results which may have been due to the detection of maternal antibodies through colostral consumption. Although studies on the length of persistence of maternal antibodies to BVDV have not been published, it is much less likely that colostral antibodies would be detected among animals >6 months, which might explain why twice as many studies were carried out in this age category compared to calves aged ≤6 months (Table 4).

The multivariate model revealed that the factors “country” and “vaccination” are essential for BVDV prevalences at animal level (i.e., PI, VI, and AB), whereas a large proportion of the variance is explained by country-specific conditions. Country-specific conditions are associated with the BVDV programmes implemented that influence the level of BVDV prevalence through the programme design itself. In contrast to AB prevalences, the factor “period” is an important factor for PI prevalences at animal and herd level. This may also be partly related to the decrease in the prevalence of PI animals over time due to eradication programmes (Fig. 4). We assumed that the factor “period” would have been essential to explain variance in the AB prevalences if more long-term studies had been incorporated into the present meta-analysis. A reduction in AB prevalence in the remainder of the herd after the removal of PI animals takes several years^18^. However, in the present study, the calculated BVDV mean pooled prevalences are mainly based on short-term studies (i.e., studies provided BVDV prevalences for less than two years for the same study herd). In contrast to PI and VI prevalences, the number of tested animals and herds can explain a high variance of AB prevalence data which may be partly related to how many different cattle in specific age categories contributed to bulk milk samples, which influenced the range of the published AB prevalence data (see 95% confidence intervals in the Supplementary Figs S1–S3). At the herd level, diagnostic methods were not identified as an essential factor to explain the variance in the data. This may explain that more common detection methods were used, compared to those at the animal level, as the basis for diagnostics regarding BVDV exposure such as antibody detection in bulk milk or targeted sampling (e.g., samples of animals in a certain age group). It was to some extent surprising that the factor “age” was identified as irrelevant in explaining the variance in PI and AB prevalence data, which might be due to the broadness of our age classification.

The multivariate model was found to be suitable to describe the PI prevalences at both animal (R^2^ = 55.6%) and herd level (R^2^ = 40.1%), as well as the AB prevalence at herd level (R^2^ = 58.9%), as the goodness-of-fit of this model was higher than that determined at AB animal level (R^2^ = 26.2%). To increase the variance explained by the factors and to determine their impact on the level of reported prevalences incurred by BVDV infection, it is desirable to have additional information on covariates. These could include management factors such as housing systems^14^, community pasturing activities^11^, artificial insemination^43^, the period when animals were enrolled in BVDV programmes^7^, mortality rate^15^ (e.g., influences the transmission rate in the herd due to the reduced number of excreting animals)^44^, lactation and pregnancy status^16^, sex^45^, sensitivity and specificity of diagnostic methods^10^, BVDV genotypes and strains^33,46^, herd demographical structure^11,43^ such as herd size, cattle density and historical herd information such as frequency of purchase and trading activities^30,47^. Although we assume that some of this information is indirectly included in the factor “country”, a limitation of our meta-analysis is that not all of these covariates, and other factors such as randomisation of tested animals in the studies, could be taken into account. All these may be explained the identified heterogeneity across the studies and may have influenced our temporal predictions of BVDV prevalences. Knowledge of these factors is considered vital to explain the difference in recorded BVDV prevalences between studies, but most veterinary studies neglect to include this important information. For instance, data on trade activities, such as the purchase of BVDV-infected animals or pregnant animals carrying a PI foetus, influence the recorded BVDV prevalence of a country as purchased animals are not representative for the analysed geographical area^17^. Only 24 studies (7.38%) provided information on the purchase and trading activities of the farms included with respect to BVDV infection. A further issue, which was identified during our systematic review, is that all the studies analysed here used the term “prevalence”, but in some studies, in particular those assessing control and/or eradication programmes testing newborn calves, incidences rather than prevalences were determined. In future, we would recommend a more accurate use of epidemiological terminology to aid comparison and possibly reduce heterogeneity between studies. However, the impact of these studies on our meta-analysis is considered to be low due to consideration of the tested population size in the variance estimations by using the double arcsine transformation i.e., the weighting of the studies in our meta-analysis based on the number of tested population size in order to minimize the heterogeneity within and between the studies due to variation in population characteristics etc.

The following limitations were identified in our meta-analysis: our analysis did not incorporate interactions between all factors because datasets for some factors were too sparse. To avoid a majorly unbalanced number of studies, we chose a very broad definition of subgroup classifications, which made the interpretation of the results somewhat difficult (e.g., age classification; Tables 2–4). In general, subgroups with a low number of studies should be interpreted with caution due to imprecise estimates. The unbalanced number of studies in some subgroups may also have contributed to the identified heterogeneity and publication bias presented here. The lack of studies and/or the unbalanced number of studies may have been caused by (i) BVDV infections that were not reported, (ii) no testing system was in place due to low number of cattle or a low level of priority to control BVDV compared to other animal diseases, as the virus has no zoonotic potential, (iii) prevalence data are available but they are not in the public domain. However, it is also possible that we may have (iv) excluded studies due to predefined exclusion criteria, or (v) studies may not have been identified by the chosen database and search terms. Given the large number of countries and studies included in our meta-analysis, the assumption has been made that the mean pooled BVDV prevalences reported here are comparable to those of countries with missing data. This assumption should be verified by sampling in these geographical regions. A further limitation is that apparent prevalence estimates were not corrected for the varying levels of sensitivity and specificity of the diagnostic tests used. The mean pooled BVDV prevalences of this study might have been different if the accuracy of applied diagnostic methods had been included. Approximately 13.85% of all studies provided information on the performance of the diagnostic tests that would allow for the calculation of the true prevalences. The determination of clinical signs may accompany diseases other than BVDV and might also influence the associated estimates of pooled prevalences. Although BVDV genotype 2 has been reported to be more virulent than BVDV genotype 1^48^, we could not identify a relationship between the clinical signs vs. non-clinical signs and the different genotypes and strains, primarily due to an insufficient number of studies assessing this aspect of the disease. Additionally, high virulence has since been determined to apply to only a small number of BVDV-2 strains^49^. An overview of the presence and frequency of the distribution of BVDV genotypes per UN region are provided by Yeşilbağ *et al*.^50^.

The strengths of the meta-analysis presented here are that it provides a generalised overview of BVDV prevalences worldwide, shows different levels of prevalences both within, and between, countries (see Supplementary Figs S1–S3), demonstrates the success of control and/or eradication programmes with regard to the reduction of BVDV prevalences (Fig. 4) and determines sources of heterogeneity between studies highlighted by (i) a wide range between BVDV prevalence data within a stratified analysis and (ii) associated data gaps of specific influencing factors (e.g., indicated by a low number of studies in some sub-groups). Despite the existence of such data restrictions, it is desirable to pool BVDV prevalences from different countries as it provides a more general and large-scale overview of study outcomes than any individual study could provide. Nevertheless, more standardised epidemiological studies are needed to address these information gaps and to provide more robust conclusions for policy makers and veterinarians regarding influencing factors on BVDV prevalences and to support the planning of future intervention efforts, such as possible restrictions on live trade to reduce the burden of this non-globally-regulated disease on the cattle population. In particular, it is important to note that countries that did not use vaccination, and control and/or eradication programmes had a higher mean pooled PI prevalence than those using such mitigation strategies.

## Methods

### Literature review and data source

A systematic review was conducted to identify studies focusing on BVDV prevalences i.e., PI and/or VI and/or AB-positive animals/herds. Three online databases were searched: ISI Web of Knowledge (covering publications from 1900 until October 2016), PubMed (from 1879 until October 2016) and Scopus (from 1960 until October 2016). We used the following predefined search term combinations: (BVDV OR BVD OR bovine viral diarrhoea virus OR bovine viral diarrhea virus) AND (PI OR persistently infected OR TI OR transiently infected OR infection) AND (Bulk milk OR serum OR blood OR ear tag) AND (Prevalence OR freedom from disease). Articles returned by the three online databases were screened in full by four reviewers (BS, FR, VR, CF). Studies that covered BVDV prevalences were then reviewed in full by one reviewer (BS) and were reviewed again for validation by two reviewers (FR, VR). The reference lists of the relevant primary articles were screened for further pertinent studies. Where uncertainties existed regarding the inclusion of studies, these were discussed between all reviewers and the corresponding author until a consensus was reached. The criteria for study inclusion were (i) focus on BVDV infection of cattle, (ii) reporting prevalence data as percentage and/or the total number of tested and positively tested animals/herds, (iii) only original studies on BVDV prevalence data, (iv) publication of the applied diagnostic method, (v) reporting of geographical area, (vi) consideration of more than one herd, (vii) use of samples from living or dead animals, except foetuses and/or aborted material, and (viii) non-modelling studies. The studies were reviewed in accordance with the predefined inclusion criteria provided in Table 1 and the data collected were entered into a Microsoft Excel datasheet. The number of studies identified and the exclusion procedure of the systematic review, in accordance with the PRISMA guidelines (Preferred Reporting Items for Systematic Reviews and Meta-Analysis), are illustrated in the flow diagram in Fig. 1. Please note that an article was divided into sub-studies if the study covered, e.g., different sampling periods, geographical regions of a country or used a combination of diagnostic methods.

### Data analysis

Weighted meta-analyses were performed using a random effect model to estimate the pooled prevalences of BVDV positive animals and herds, by pooling data from studies deemed eligible. In contrast to a fixed-effect model, the random procedure incorporated an extra variance component, to find variability between the studies (heterogeneity) in addition to within-study (sampling) variance^51^ as a result of sampling error. A stratified analysis of the possible source of heterogeneity was carried out including the factors in Table 1 (subgroup analysis). Direct weighting by sample size is less suitable due to substantial differences in the study size, thus a weighted measure was used according to the Paule and Mandel Method based on the formula w_i_ = 1/(v_i_ + τ²)^52,53^ considering the sample size indirectly via the variance. In detail, the mean prevalences were weighted (w_i_) based on the inverse of within-study variance (v_i_) and by the variability across the studies (τ²). Freeman-Tukey double arcsine transformation^54^ was applied for variance-stabilising of the BVDV prevalence data distribution. The corresponding back-transformation was performed according to the equations described by Miller^55^. The degree of between-study variance was determined using the Cochran’s Q-test (P < 0.05 indicated heterogeneity). The Higgins inverse variance (I^2^) index (I^2^ > 50% indicated heterogeneity) was calculated to determine the percentage of total variation in effect estimates across the BVDV prevalence studies as a result of heterogeneity rather than chance^56^. A sensitivity analysis was performed to determine publication bias by inspection of funnel plots^57^, conducting a regression test for funnel plot asymmetry and performing an influential case diagnostic (e.g., calculating DfBeta^58^, Cook’s distance^59^). The sensitivity analysis determined nine outliers, which were excluded from the presented meta-analyses (Supplementary Fig. S4). The weight contribution proportion of each study to the meta-analyses and the pooled prevalences bounded by 95% confidence intervals (CIs) are shown in the Forest Plots, presented as Supplementary Figures S1–S3. The temporal development of BVDV prevalences per UN region was predicted until 2020. To avoid imprecise predictions, UN regions incorporating less than 15 sub-studies were excluded from the temporal trend analyses.

Univariate meta-regression analyses were conducted to identify factors according to Table 1 that may have had a significant impact on the level of reported BVDV infection prevalences. A two-step selection process of variables was carried out. First, deletion of variables was carried out, based on the results of collinearity assessment using the Godman-Kruskal’s tau and Wald test of logistic regression. Non-correlated variables with a p-value cut-off point lower than 0.25 were considered in the meta-regression analyses^60^. The explainable proportion of these factors (R^2^) on the variability of recorded prevalences was determined. Second, the remaining variables were deleted, based on the values of Akaike Information Criteria, corrected for small sample size (AICc) and a p-value cut-off point higher than 0.1. Factors not altering the R^2^ by more than 10% of the full model were deleted to include only the most relevant factors without losing model-fit-accuracy. The meta-analyses were implemented in R (Version 3.4.1R Foundation for Statistical Computing, Vienna, Austria) using the Metafor package^61^. Pooled BVDV prevalences per individual country were illustrated in maps which were created using QGIS 2.18.13 (Quantum GIS GmbH, US).

## Electronic supplementary material

Supplementary Material

## Data Availability

The generated datasets and analyses of the present study are available from the corresponding author upon request.
