# Supplementary material for: A meta-analysis of bovine viral diarrhoea virus (BVDV) prevalences in the global cattle population
Source: Sci Rep. 2018 Sep 26;8:14420. doi: 10.1038/s41598-018-32831-2 (PMC6158279; doi:10.1038/s41598-018-32831-2)
Supplement: Supplementary file 1 — Supplementary Material [file 41598_2018_32831_MOESM1_ESM.docx]

Supplementary Material

A meta-analysis of bovine viral diarrhoea virus (BVDV) prevalences in the global cattle population

Bettina Scharnböck^a^#, Franz-Ferdinand Roch^a^#, Veronika Richter^a^, Carsten Funke^a,b^, Clair L. Firth^a^, Walter Obritzhauser^a^, Walter Baumgartner^c^, Annemarie Käsbohrer^a,d^, Beate Pinior^a^*

^a^ Institute for Veterinary Public Health, University of Veterinary Medicine Vienna, Veterinärplatz 1, 1210 Vienna, Austria

^b^ Institute of Veterinary Pathology, Justus-Liebig-University, Frankfurter Straße 96, 35392 Giessen, Germany

^c^ University Clinic for Ruminants, University of Veterinary Medicine Vienna, Veterinärplatz 1, 1210 Vienna, Austria

^d^ Department of Biological Safety, Federal Institute for Risk Assessment (BfR), Diedersdorfer Weg 1, 12277 Berlin, Germany

# Both authors contributed equally to this work.

* Corresponding author. Tel.: +43 125 077 3505.

E-mail address: [Beate.Pinior@vetmeduni.ac.at](mailto:Beate.Pinior@fli.bund.de) (B. Pinior)

**Supplementary data**

Fig. S1. Forest plot of PI studies at animal level ordered by UN region and publication year. N.B. the publication year and sampling period deviate from each other on average by 3.6 years.

Fig. S2. Forest plot of VI studies at animal level ordered by UN region and publication year. N.B. the publication year and sampling period deviate from each other on average by 4.3 years.

Fig. S3. Forest plot of AB-positive studies at animal level ordered by UN region and publication year. N.B. the publication year and sampling period deviate from each other on average by 4.2 years.

Fig. S4. Sensitivity analysis and the identified outliers (shown as red circles). a) Outliers at PI animal level; b) Outliers at VI animal level; c) Outliers at PI herd level; d) Outliers at VI herd level. N.B. no outliers were identified for AB-positive animals and herds.

Table S1. Meta-analysis of studies reporting the prevalence of PI herds.

Table S2. Meta-analysis of studies reporting the prevalence of VI herds.

Table S3. Meta-analysis of studies reporting the prevalence of AB-positive herds.

Fig. S1. Forest plot of PI studies at animal level ordered by UN region and publication year. N.B. the publication year and sampling period deviate from each other on average by 3.6 years.


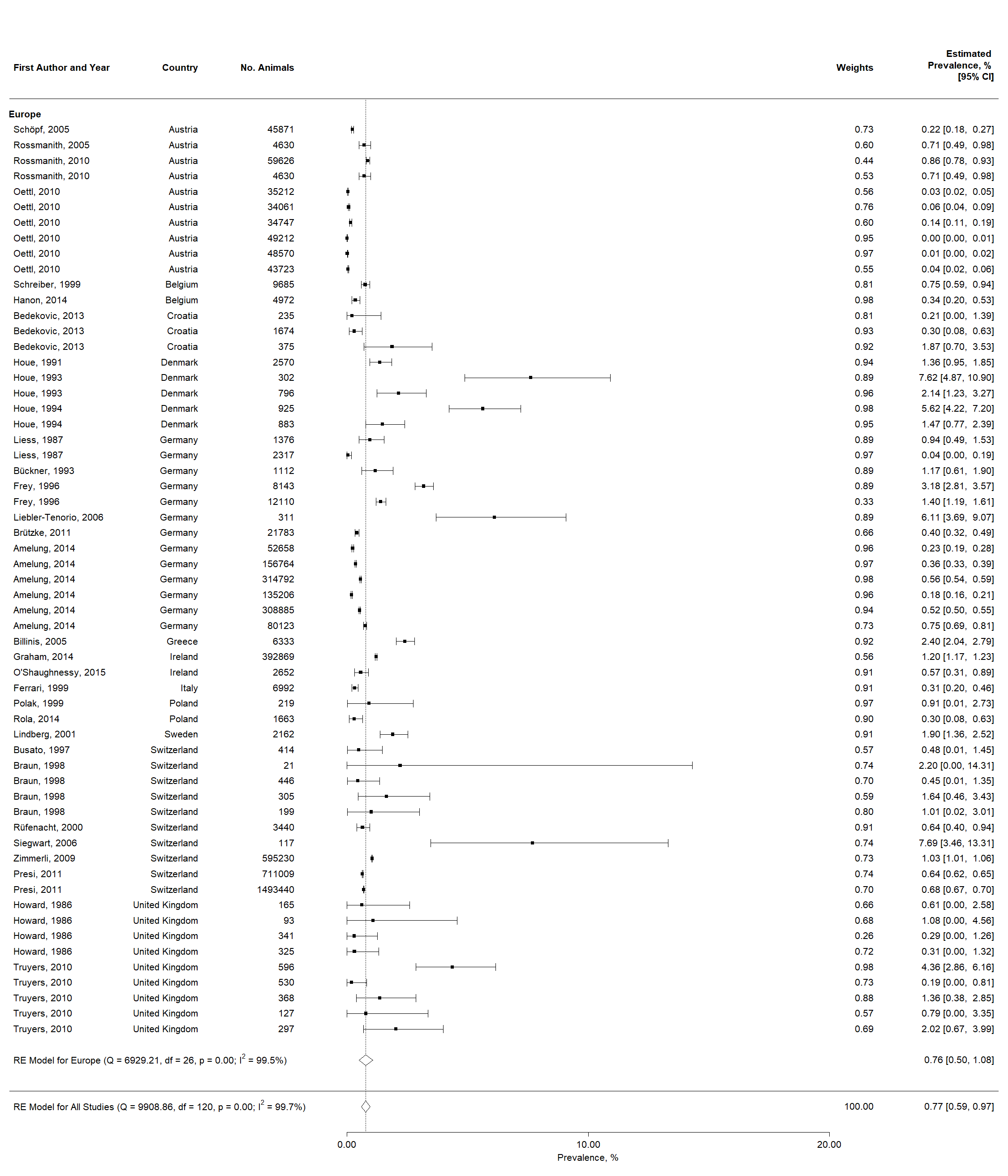


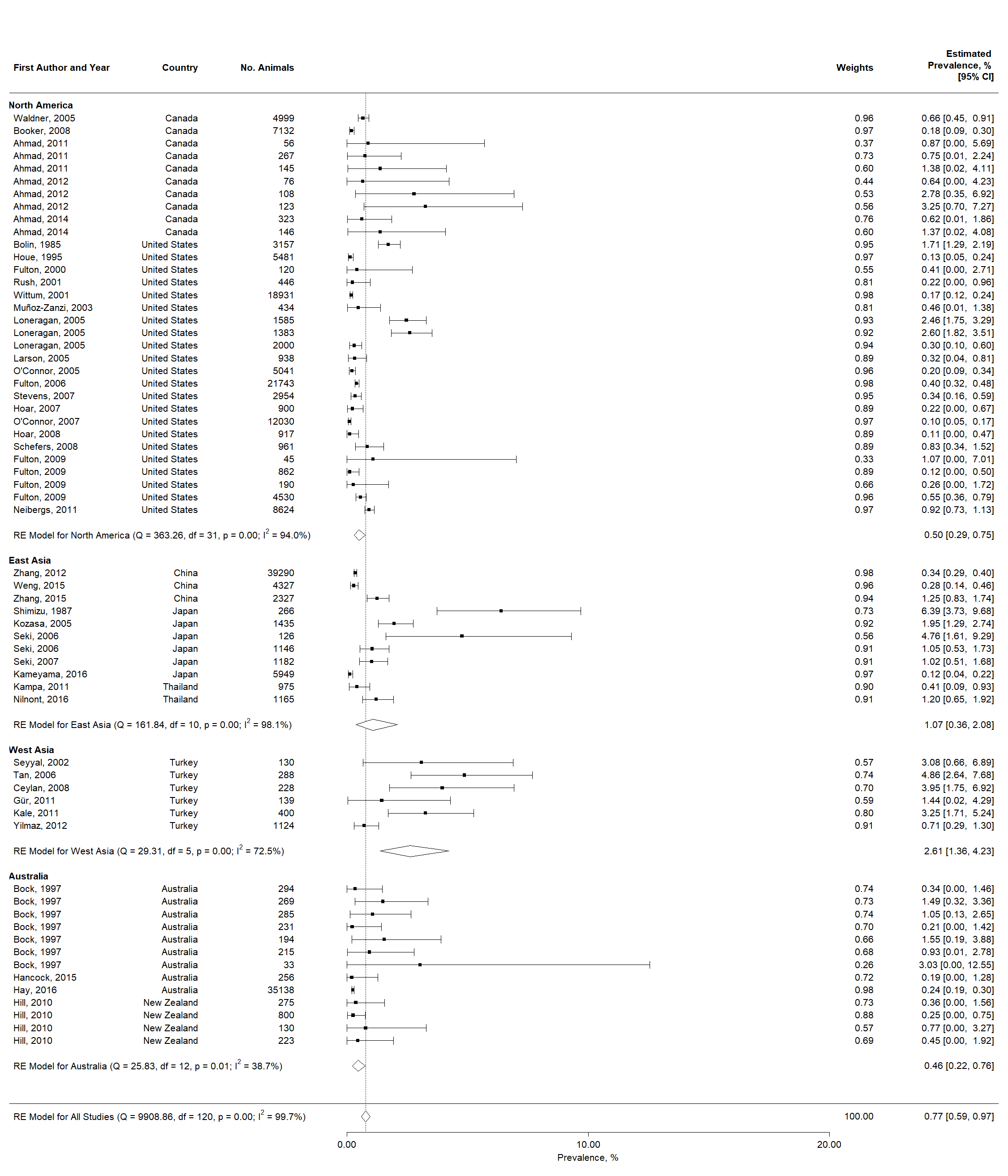


Fig. S2. Forest plot of VI studies at animal level ordered by UN region and publication year. N.B. the publication year and sampling period deviate from each other on average by 4.3 years.


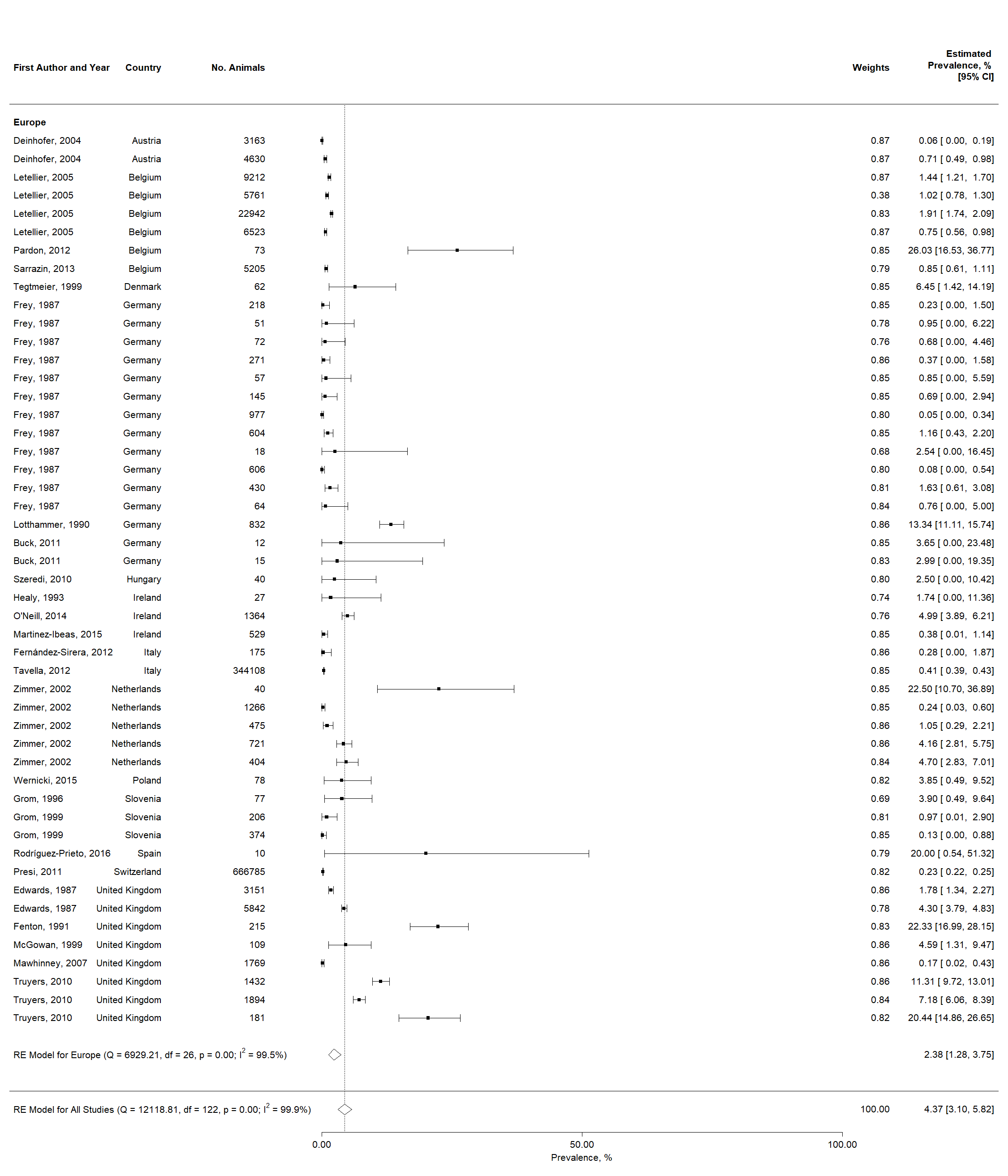


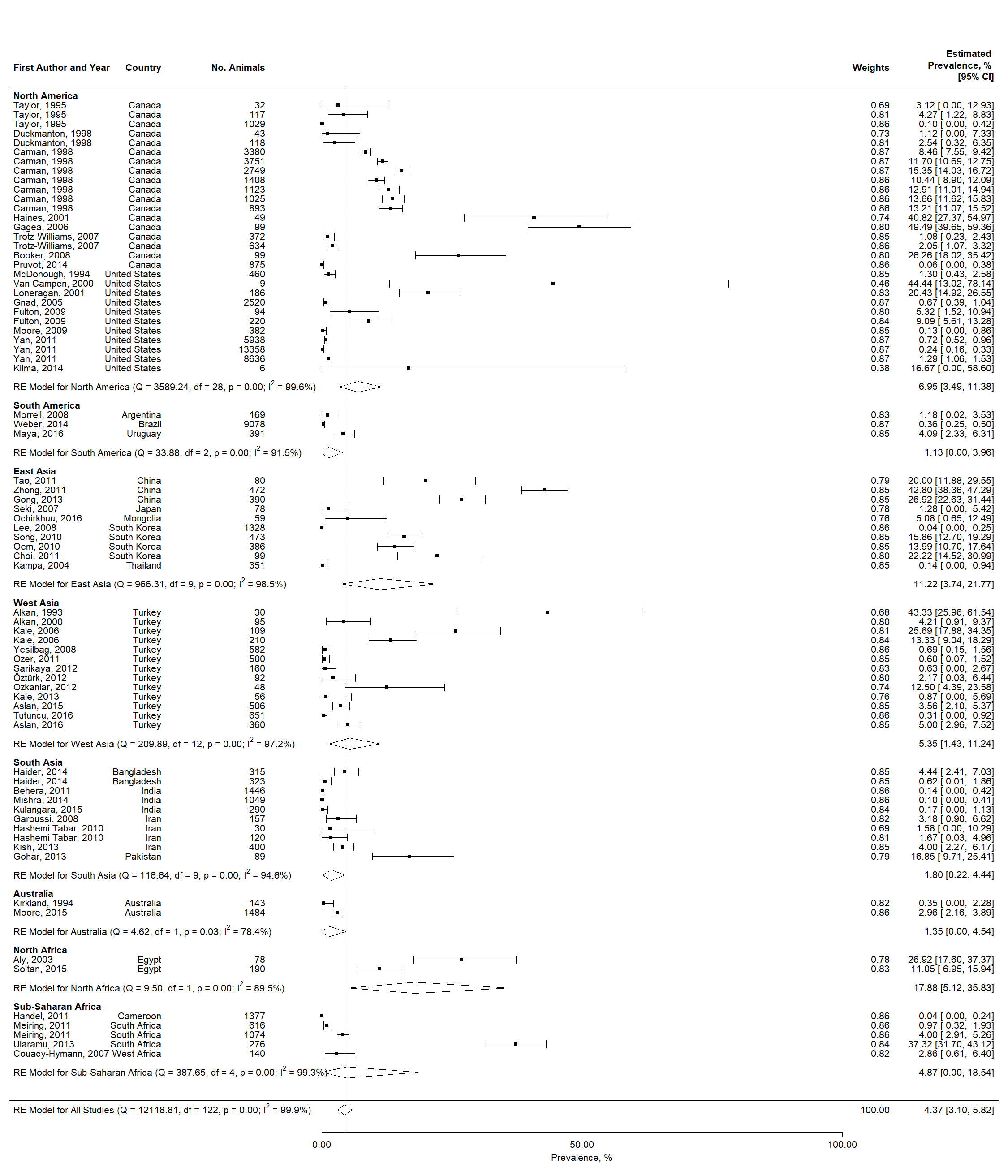


Fig. S3. Forest plot of AB-positive studies at animal level ordered by UN region and publication year. N.B. the publication year and sampling period deviate from each other on average by 4.2 years.


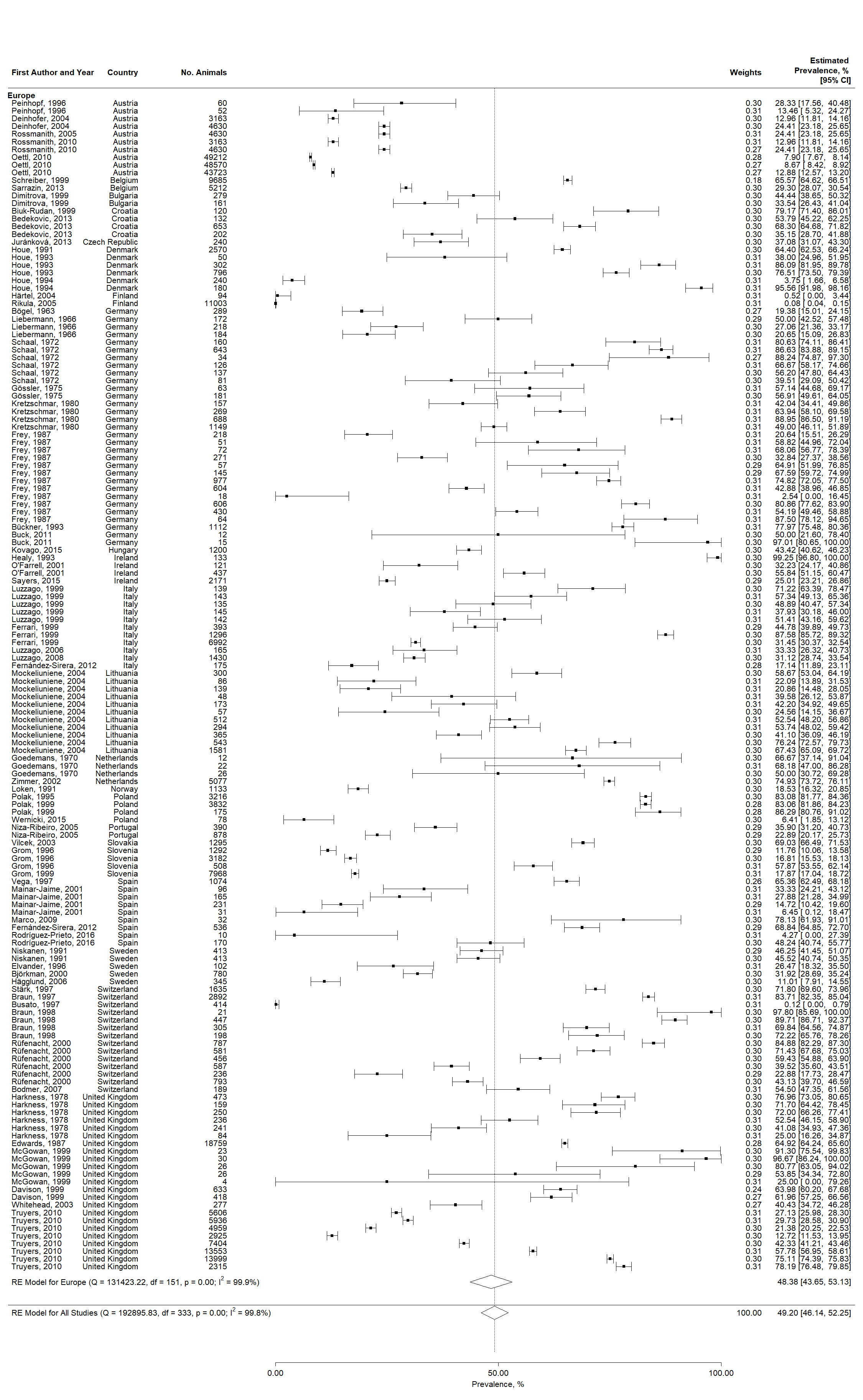


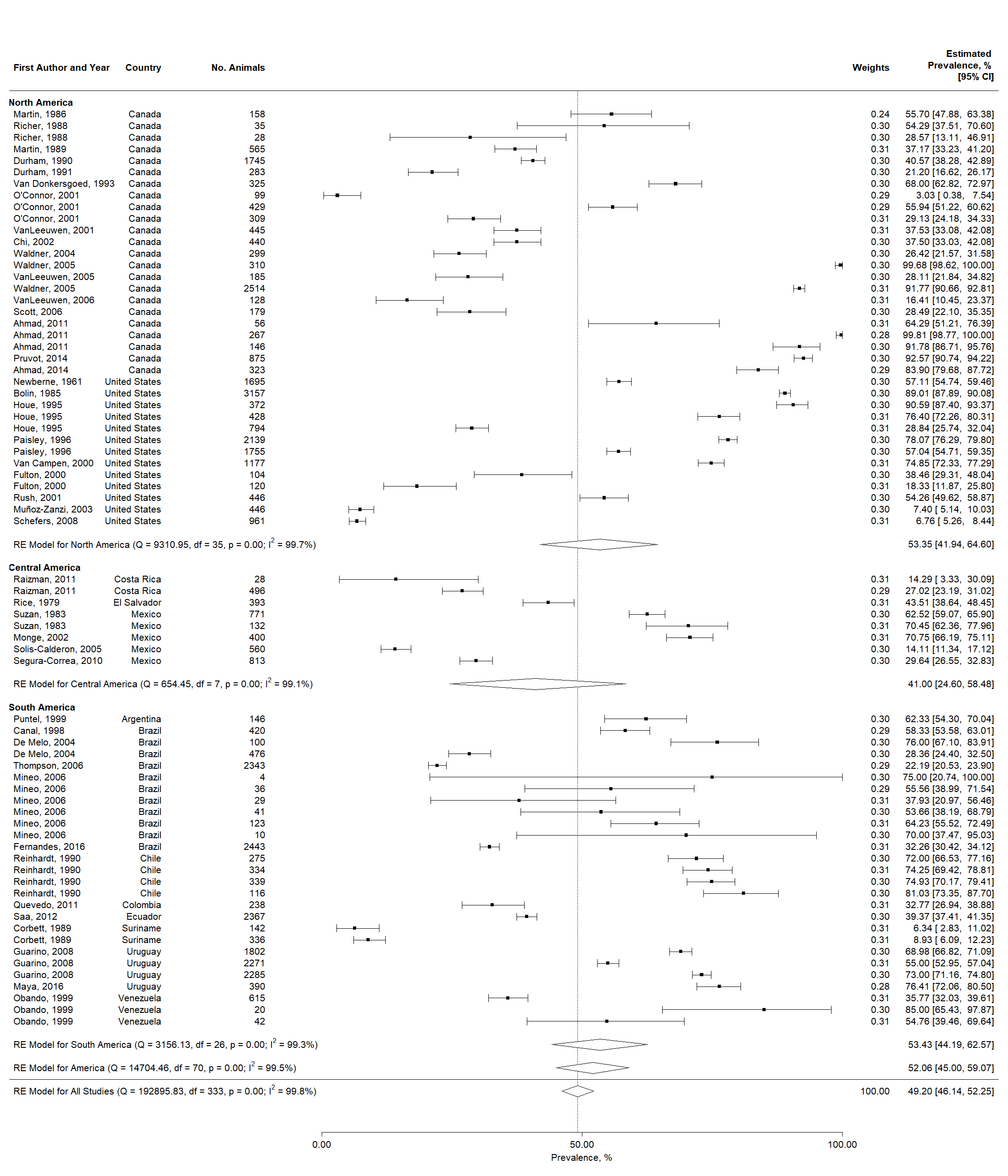


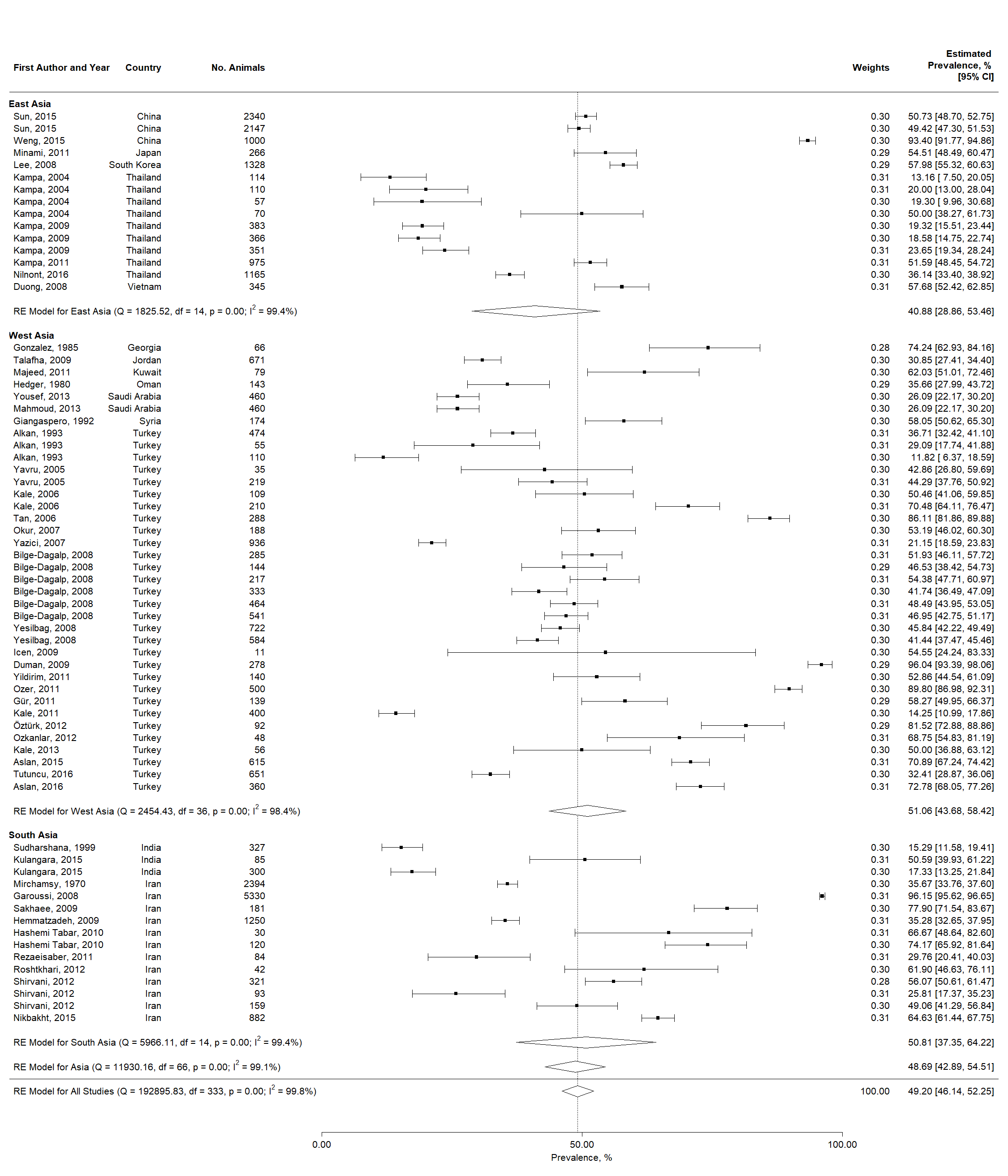


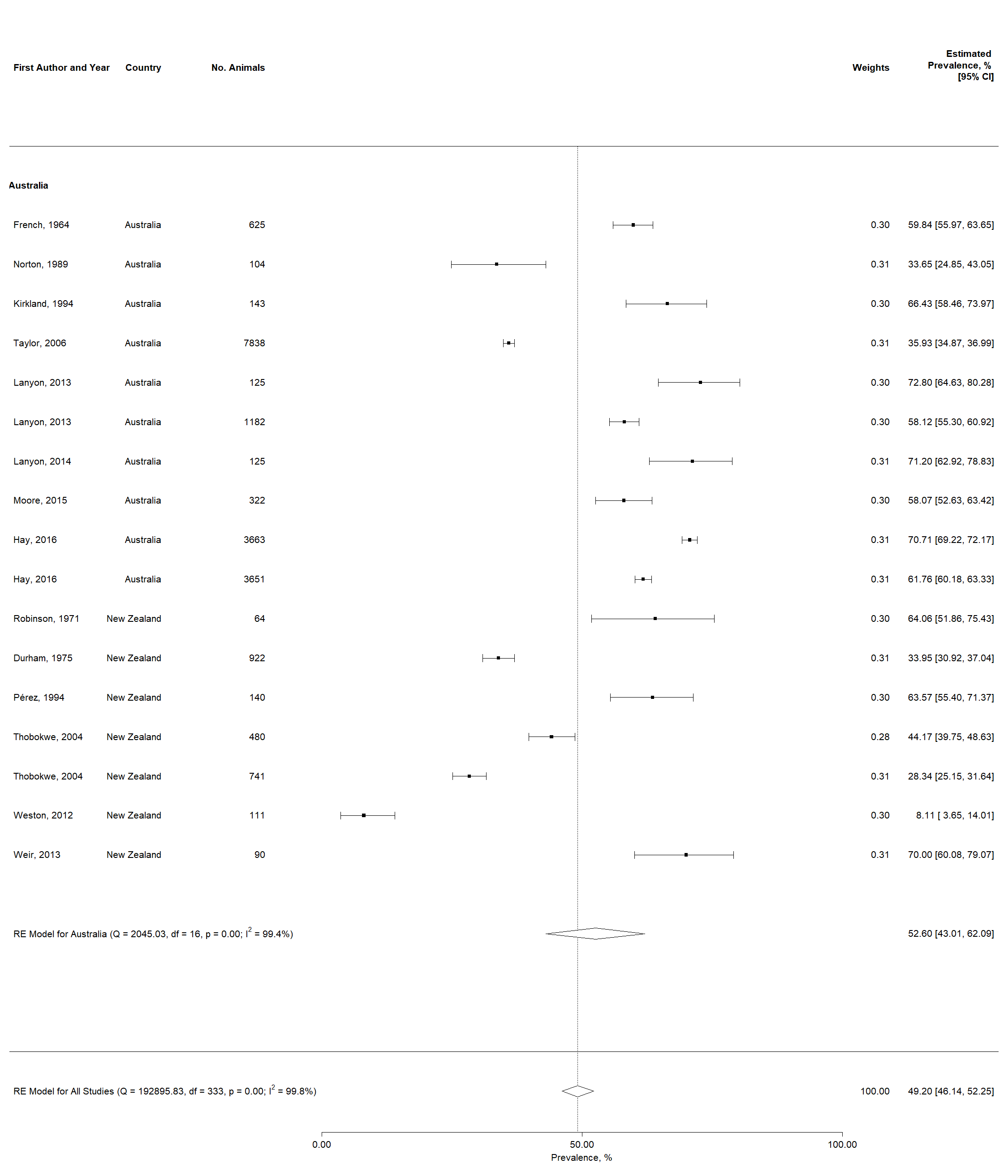


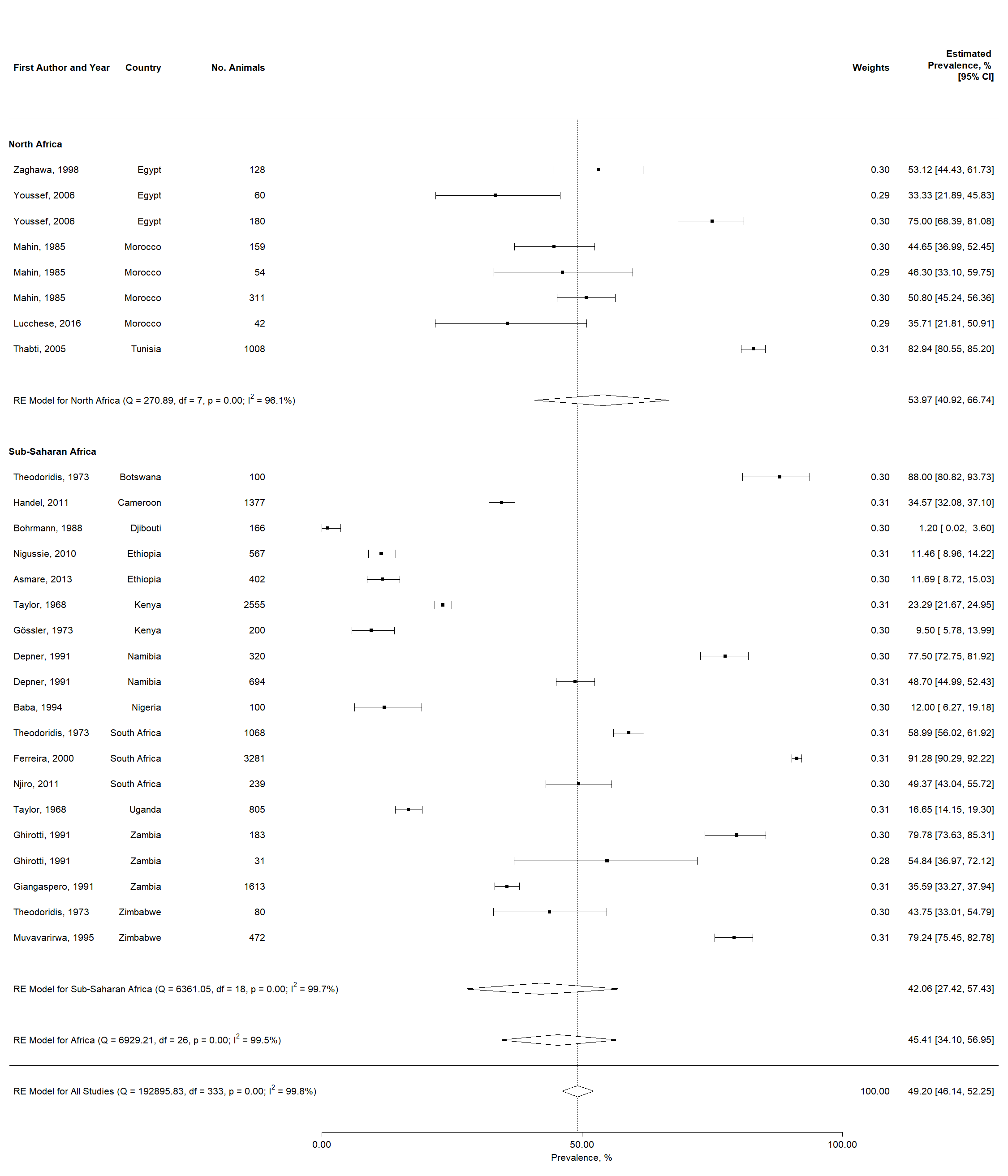


Fig. S4. Sensitivity analysis and the identified outliers (shown as red circles). a) Outliers at PI animal level; b) Outliers at VI animal level; c) Outliers at PI herd level; d) Outliers at VI herd level. N.B. no outliers were identified for AB-positive animals and herds.


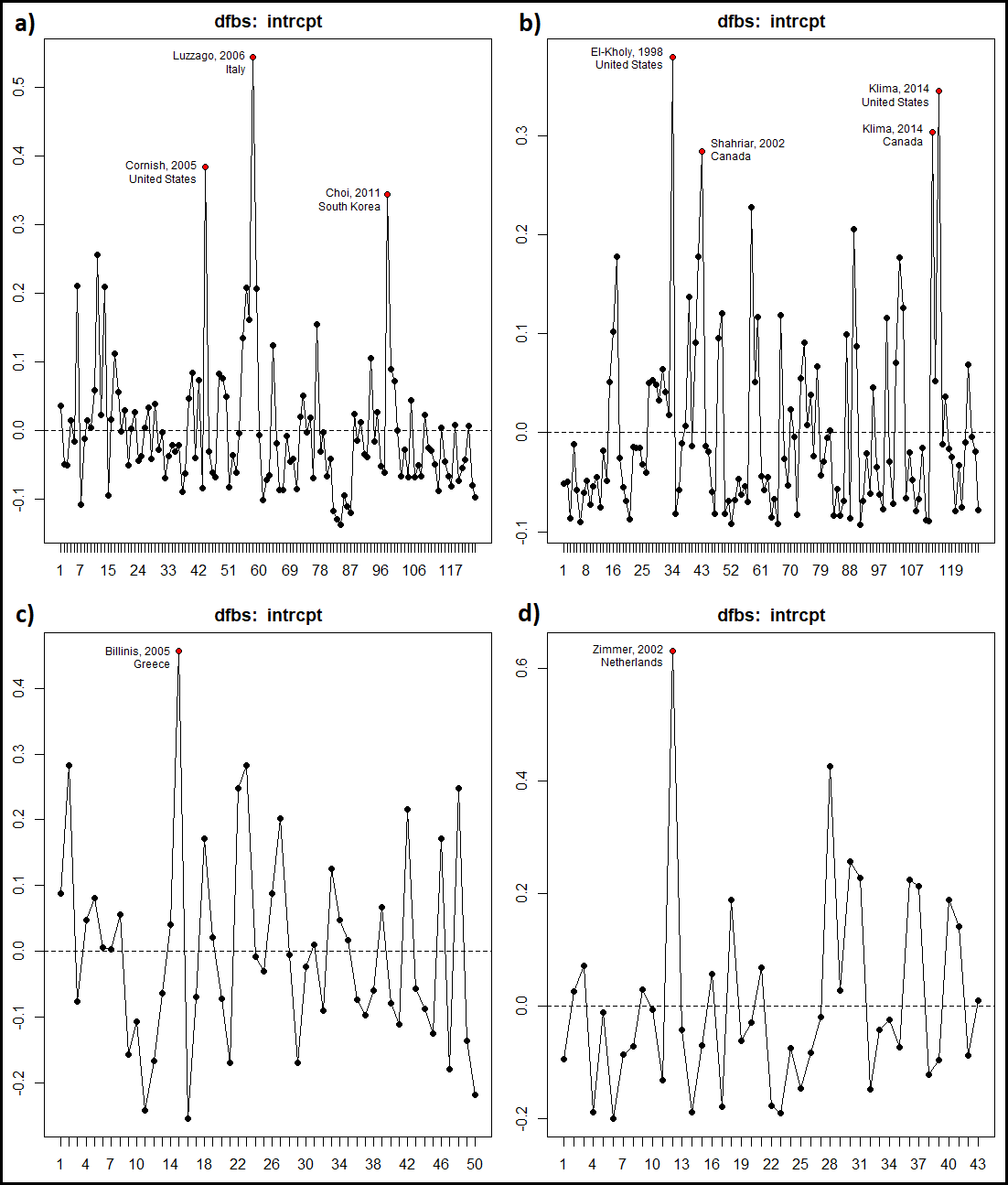


Table S1. Meta-analysis of studies reporting the prevalence of PI herds.

|  | No. of prevalence inputs | | Sample size (No. herds) | Weighted mean estimate | Confidence Interval (95%) | I² (%) | |
| --- | --- | --- | --- | --- | --- | --- | --- |
| Overall | | 49 | 43,695 | 27.23 | (18.91-36.32) | | 99.6 |
| Subgroup^a^ | |  |  |  |  | |  |
| UN Region | |  |  |  |  | |  |
| Europe | | 20 | 42,349 | 24.18 | (13.30-36.75) | | 99.79 |
| North America | | 13 | 671 | 22.64 | (11.79-35.39) | | 89.05 |
| East Asia | | 10 | 476 | 38.84 | (15.01-65.50) | | 95.92 |
| West Asia | | 3 | 132 | 23.18 | (0.00-85.03) | | 93.84 |
| Australia | | 3 | 67 | 34.74 | (0.00-89.20) | | 95.27 |
| Period | |  |  |  |  | |  |
| 1988-1991 | | 2 | 40 | 42.36 | (23.91-61.83) | | 30.19 |
| 1992-2001 | | 11 | 5,382 | 20.48 | (11.39-31.09) | | 95.52 |
| 2002-2016 | | 20 | 37,725 | 18.88 | (8.79-31.17) | | 99.77 |
| Not specified | | 16 | 548 | 45.55 | (25.51-66.26) | | 93.93 |
| Production system | |  |  |  |  | |  |
| Beef | | 11 | 12,687 | 18.73 | (6.15-35.11) | | 97.56 |
| Dairy | | 24 | 18,222 | 34.57 | (20.25-50.29) | | 97.46 |
| Mixed | | 10 | 5,235 | 20.09 | (8.80-33.96) | | 97.13 |
| Not specified | | 4 | 7,551 | 36.74 | (8.74-70.04) | | 93.68 |
| Age group | |  |  |  |  | |  |
| ≤6 months | | 12 | 7,868 | 22.39 | (10.03-37.37) | | 93.97 |
| >6 months | | 3 | 223 | 14.66 | (0.00-54.63) | | 96.95 |
| Mixed | | 6 | 389 | 32.57 | (12.01-56.83) | | 93.39 |
| Not specified | | 28 | 35,215 | 29.30 | (17.24-42.81) | | 99.74 |
| Vaccination | |  |  |  |  | |  |
| Yes | | 13 | 687 | 29.57 | (18.47-41.86) | | 88.57 |
| No | | 16 | 17,086 | 15.66 | (4.90-29.94) | | 99.43 |
| Not specified | | 20 | 25,922 | 35.65 | (20.19-52.59) | | 99.68 |
| Clinical signs | |  |  |  |  | |  |
| Yes | | 6 | 119 | 44.52 | (14.34-76.83) | | 90.54 |
| No | | 6 | 673 | 29.35 | (4.68-62.08) | | 98.33 |
| Not specified | | 37 | 42,903 | 24.35 | (15.75-33.96) | | 99.64 |
| Programme | |  |  |  |  | |  |
| Yes | | 12 | 41,940 | 20.89 | (6.97-38.77) | | 99.91 |
| No | | 1^b^ | 35 | 18.83 | (6.63-34.49) | | - |
| Not specified | | 36 | 1,720 | 30.06 | (19.93-41.12) | | 94.54 |
| Diagnostic method | |  |  |  |  | |  |
| Direct detection method | | 1 | 38 | 36.29 | (20.93-53.02) | | - |
| RT-PCR | | 7 | 322 | 36.14 | (11.38-65.03) | | 92.09 |
| AG ELISA | | 18 | 13,140 | 20.41 | (11.20-31.18) | | 98.96 |
| Cell culture-based system | | 8 | 17,521 | 23.06 | (4.11-49.26) | | 97.04 |
| Mixed | | 15 | 12,674 | 35.75 | (17.50-56.12) | | 97.87 |

^a^ All p-values except for the period 1988-1991 (p=0.23), no BVDV programmes (p= could not be determined) and direct detection method (p= could not be determined) were ≤ 0.01 and were calculated using the Cochran’s Q-test. The regression test for funnel plot asymmetry was z=6.01; p<0.0001.

^b^ Sub-groups with a low number of studies should be interpretated with caution due to imprecise estimates of pooled BVDV prevalences.

Table S2. Meta-analysis of studies reporting the prevalence of VI herds.

|  | No. of prevalence inputs | | | Sample size (No. herds) | | Weighted mean estimate | | Confidence Interval  (95%) | | I² (%) |
| --- | --- | --- | --- | --- | --- | --- | --- | --- | --- | --- |
| Overall | | 42 | 50,748 | | 17.80 | | (11.51-24.97) | | 99.10 | |
| Subgroup^a^ | |  |  | |  | |  | |  | |
| UN Region | |  |  | |  | |  | |  | |
| Europe | | 22 | 48,951 | | 12.93 | | (7.93-18.76) | | 99.01 | |
| North America | | 6 | 1,050 | | 6.84 | | (1.33-15.04) | | 91.78 | |
| South America | | 1 | 346 | | 5.07 | | (2.61-8.08) | | - | |
| East Asia | | 2 | 64 | | 37.66 | | (0.00-100.00) | | 98.33 | |
| West Asia | | 2 | 30 | | 49.25 | | (12.87-86.07) | | 77.84 | |
| South Asia | | 4 | 53 | | 38.67 | | (9.39-72.91) | | 83.19 | |
| Australia | | 3 | 250 | | 22.05 | | (0.57-57.25) | | 95.83 | |
| North Africa | | 2 | 4 | | 77.44 | | (40.06-99.72) | | 0 | |
| Period | |  |  | |  | |  | |  | |
| 1985-1991 | | 2 | 104 | | 27.13 | | (18.58-36.53) | | 0 | |
| 1992-2001 | | 5 | 607 | | 11.79 | | (0.00-35.36) | | 97.15 | |
| 2002-2016 | | 21 | 49,342 | | 16.45 | | (7.80-27.12) | | 99.67 | |
| Not specified | | 14 | 695 | | 21.11 | | (10.27-34.20) | | 91.56 | |
| Production system | |  |  | |  | |  | |  | |
| Beef | | 7 | 1,634 | | 19.59 | | (5.73-38.12) | | 96.37 | |
| Dairy | | 20 | 5,094 | | 14.92 | | (6.52-25.52) | | 98.11 | |
| Mixed | | 5 | 39,179 | | 10.09 | | (4.89-16.55) | | 95.16 | |
| Not specified | | 10 | 4,841 | | 28.34 | | (12.01-47.88) | | 99.18 | |
| Age group | |  |  | |  | |  | |  | |
| ≤6 months | | 2 | 20 | | 87.13 | | (68.94-98.62) | | - | |
| >6 months | | 5 | 1,274 | | 13.51 | | (1.00-34.00) | | 97.85 | |
| Mixed | | 9 | 42,629 | | 21.41 | | (8.46-37.68) | | 99.61 | |
| Not specified | | 26 | 6,825 | | 13.94 | | (7.87-21.15) | | 97.56 | |
| Vaccination | |  |  | |  | |  | |  | |
| Yes | | 9 | 4,852 | | 10.85 | | (3.54-20.74) | | 98.16 | |
| No | | 12 | 38,390 | | 31.43 | | (12.96-53.22) | | 97.22 | |
| Not specified | | 21 | 7,506 | | 14.68 | | (8.21-22.39) | | 98.03 | |
| Clinical signs | |  |  | |  | |  | |  | |
| Yes | | 6 | 186 | | 49.24 | | (23.39-75.29) | | 88.83 | |
| No | | 3 | 112 | | 8.89 | | (0.00-36.01) | | 85.29 | |
| Not specified | | 33 | 50,450 | | 14.62 | | (8.99-21.16) | | 99.25 | |
| Programme | |  |  | |  | |  | |  | |
| Yes | | 13 | 46,638 | | 18.24 | | (10.49-27.33) | | 99.38 | |
| No | | 3 | 147 | | 38.32 | | (3.53-81.63) | | 95.55 | |
| Not specified | | 26 | 3,963 | | 15.31 | | (7.54-24.84) | | 97.59 | |
| Diagnostic method | |  |  | |  | |  | |  | |
| Direct detection method | | 3 | 127 | | 13.02 | | (5.37-22.83) | | 38.76 | |
| RT-PCR | | 16 | 2,620 | | 12.53 | | (3.44-25.19) | | 97.89 | |
| AG ELISA | | 10 | 1,327 | | 28.53 | | (14.10-45.34) | | 95.84 | |
| Cell culture-based system | | 1^b^ | 250 | | 0.18 | | (0.00-1.83) | | - | |
| Mixed | | 12 | 46,424 | | 20.08 | | (10.38-31.68) | | 99.59 | |

^a^ All p-values except for West Asia (p=0.03), North Africa (p=1.00), East Asia and South America (p=could not be determined), period 1985-1991 (p=0.48), age group < 6 months (p= could not be determined), clinical signs (p=0.08), direct detection method (p=0.22) and cell culture based system (p= could not be determined) were ≤ 0.01 and were calculated using the Cochran’s Q-test. The regression test for funnel plot asymmetry was z=5.40; p<0.0001.

^b^ Sub-groups with a low number of studies should be interpretated with caution due to imprecise estimates of pooled BVDV prevalences.

Table S3. Meta-analysis of studies reporting the prevalence of AB-positive herds.

|  | No. of prevalence inputs | | | Sample size (No. herds) | | Weighted  mean estimate | | Confidence  Interval  (95%) | I² (%) | |
| --- | --- | --- | --- | --- | --- | --- | --- | --- | --- | --- |
| Overall | | 144 | 216,105 | | 67.74 | | (62.07-73.18) | | | 99.83 |
| Subgroup^a^ | |  |  | |  | |  | | |  |
| UN Region | |  |  | |  | |  | | |  |
| Europe | | 77 | 209,424 | | 60.45 | | (51.84-68.77) | | | 99.92 |
| North America | | 14 | 841 | | 66.01 | | (48.83-81.43) | | | 94.45 |
| Central America | | 4 | 133 | | 61.00 | | (35.72-83.73) | | | 86.75 |
| South America | | 18 | 2,344 | | 80.53 | | (69.35-89.89) | | | 96.49 |
| East Asia | | 9 | 1,987 | | 68.68 | | (49.58-85.20) | | | 98.13 |
| West Asia | | 7 | 213 | | 92.83 | | (84.04-98.70) | | | 52.61 |
| South Asia | | 4 | 193 | | 70.83 | | (35.77-96.40) | | | 95.32 |
| Australia | | 5 | 555 | | 91.84 | | (84.94-97.04) | | | 81.35 |
| North Africa | | 2 | 27 | | 52.46 | | (31.49-73.02) | | | 6.30 |
| Sub-Saharan Africa | | 4 | 388 | | 76.87 | | (27.35-100.00) | | | 98.68 |
| Period | |  |  | |  | |  | | |  |
| 1964-1991 | | 14 | 1,078 | | 67.01 | | (50.42-81.83) | | | 95.81 |
| 1992-2001 | | 56 | 130,957 | | 64.85 | | (55.04-74.12) | | | 99.90 |
| 2002-2016 | | 41 | 79,674 | | 66.08 | | (54.59-76.76) | | | 99.87 |
| Not specified | | 33 | 4,396 | | 75.09 | | (65.26-83.88) | | | 96.90 |
| Production system | |  |  | |  | |  | | |  |
| Beef | | 13 | 8,409 | | 64.31 | | (40.93-84.83) | | | 99.65 |
| Dairy | | 96 | 199,661 | | 68.11 | | (61.25-74.64) | | | 99.87 |
| Mixed | | 21 | 7,011 | | 64.78 | | (49.28-78.96) | | | 99.28 |
| Not specified | | 14 | 1,024 | | 72.65 | | (55.49-87.18) | | | 96.22 |
| Age group | |  |  | |  | |  | | |  |
| ≤6 months | | 5 | 28 | | 70.23 | | (35.28-96.04) | | | 74.80 |
| >6 months | | 25 | 5,938 | | 65.74 | | (51.59-78.71) | | | 98.94 |
| Mixed | | 27 | 2,163 | | 74.75 | | (60.70-86.78) | | | 97.59 |
| Not specified | | 87 | 207,976 | | 66.04 | | (58.90-72.86) | | | 99.89 |
| Vaccination | |  |  | |  | |  | | |  |
| Yes | | 10 | 990 | | 77.10 | | (60.35-90.64) | | | 95.34 |
| No | | 83 | 18,764 | | 73.73 | | (67.16-79.86) | | | 98.68 |
| Not specified | | 51 | 196,351 | | 56.07 | | (45.35-66.54) | | | 99.95 |
| Clinical signs | |  |  | |  | |  | | |  |
| Yes | | 20 | 973 | | 62.09 | | (46.05-76.99) | | | 95.00 |
| No | | 4 | 468 | | 44.26 | | (25.53-63.80) | | | 90.76 |
| Not specified | | 120 | 214,664 | | 69.38 | | (63.21-75.25) | | | 99.86 |
| Programme | |  |  | |  | |  | | |  |
| Yes | | 21 | 180,733 | | 31.56 | | (19.24-45.20) | | | 99.97 |
| No | | 12 | 3,137 | | 72.75 | | (49.95-90.98) | | | 99.34 |
| Not specified | | 111 | 32,235 | | 74.10 | | (68.72-79.18) | | | 98.83 |
| Diagnostic method | |  |  | |  | |  | | |  |
| AB ELISA | | 100 | 213,781 | | 65.25 | | (58.02-72.18) | | | 99.90 |
| NT | | 41 | 2,298 | | 72.72 | | (63.66-81.01) | | | 94.28 |
| Mixed | | 1^b^ | 4 | | 86.03 | | (45.72-100.00) | | | - |
| Other | | 2 | 22 | | 91.12 | | (62.59-100.00) | | | 46.08 |

^a^ All p-values except for North Africa (p=0.30) and diagnostic methods “other” (p=0.17) and “mixed” (p=could not be determined) were ≤ 0.01 and were calculated using the Cochran’s Q-test. The regression test for funnel plot asymmetry was z=3.01; p=0.0026.

^b^ Sub-groups with a low number of studies should be interpretated with caution due to imprecise estimates of pooled BVDV prevalences.
